# Short Wavelength Infrared Diimmonium Nano-Agents for Photoacoustic Imaging

**DOI:** 10.1038/s41467-026-78613-7

**Published:** 2026-10-12

**Authors:** Longcai Liu, Yang Chen, Liya Yu, Xiaozhou Mou, Guangxue Feng, Yu Cai

**Affiliations:** 1 Center for Rehabilitation Medicine, Rehabilitation & Sports Medicine Research Institute of Zhejiang Province, Department of Rehabilitation Medicine, Cancer Center, Zhejiang Provincial People’s Hospital (Affiliated People’s Hospital), Hangzhou Medical College, Hangzhou, Zhejiang 310014 China; 2 State Key Laboratory of Luminescent Materials and Devices, Guangdong Provincial Key Laboratory of Luminescence from Molecular Aggregates, School of Materials Science and Engineering, South China University of Technology, Guangzhou, 510640 China

**Keywords:** Imaging, Photochemistry

## Abstract

Photoacoustic imaging (PAI) as an innovative biomedical imaging modality shows high contrast and superior spatial resolution, yet remains limited by underdeveloped second near-infrared (NIR-II, 1000-1700 nm) contrast agents, particularly neglecting the NIR-IIb (1500-1700 nm) window. In this work, we designed a new class of diimmonium (DB)-based dicationic nano-agents (DB·2X, X = TFSI^-^, B(C_2_O_4_)_2_^-^, or BF_4_^-^) for 1700 nm excitation PAI. Density functional theory (DFT) calculations reveal that dicationic DB^2+^ exhibits a dramatically reduced excitation energy (1.24 eV) and enhanced oscillator strength (f = 1.11) by molecular engineering, attributed to a symmetry-allowed local excitation mechanism for electronic restructuring. Single-crystal X-ray diffraction further demonstrates the intermolecular spacings >5.0 Å in DB·2X aggregates, facilitating optimized non-radiative transitions for amplified PAI signals. In vivo study using different disease models shows that DB·2TFSI nano-agents under 1700 nm excitation can achieve higher signal-to-background ratio and minimized interference from endogenous chromophores compared to conventional 1064 nm excitation. Biocompatibility assessments confirm low cytotoxicity, underscoring potential for further development of DB·2TFSI nano-agents. This work delivers a robust molecular design paradigm for non-radiative decay optimization, and for the first time establishes 1700 nm-excitable PAI nano-agents, paving the way for tissue high-quality biomedical imaging.

## Abstract (web summary)

Photoacoustic imaging in the NIR-IIb window lacks effective contrast agents. Here, the authors develop 1700-nm-excitable diimmonium nano-agents that enhance photoacoustic contrast and reduce endogenous background, enabling high-quality in vivo imaging.

## Introduction

Photoacoustic imaging (PAI) represents a groundbreaking biomedical imaging modality that combines the optical high contrast imaging with the superior spatial resolution of ultrasound imaging, enabling non-invasive visualization of deep tissue structures with high clarity^1–3^. This technique relies on laser-induced thermoelastic expansion to generate ultrasonic waves that encode optical absorption information, effectively overcoming the fundamental balance between penetration depth and resolution inherent^4–6^. Despite its transformative potential, widespread clinical adoption of PAI faces significant challenges, particularly in optimizing excitation wavelengths to minimize photon scattering in biological tissues while maximizing signal-to-background ratios (SBR)^7–9^. Traditional near-infrared (NIR-I, 780-900 nm) contrast agents (e.g., indocyanine green)^10,11^ suffer from limited tissue penetration and strong interference from endogenous chromophores like hemoglobin^4,12–15^. The existing NIR-II (1000-1700 nm) agents are predominantly constrained to 1064 nm excitation^16,17^, leaving the clinically promising NIR-IIb (1500-1700 nm) window largely unexplored. Therefore, while preclinical studies have demonstrated the NIR-II advantages in reducing tissue scattering and suppressing autofluorescence, current contrast agents fail to fully exploit the NIR-II potential for centimeter-scale penetration with high contrast.

This limitation stems from both material design challenges in synthesizing agents with optimal 1500-1700 nm absorption and a fundamental lack of understanding of molecular engineering principles for this spectral range^18–21^. The current strategy for developing NIR-II molecules involves constructing extended planar conjugated frameworks or strong intramolecular donor-acceptor (D-A) interactions^22,23^. Benzothiadiazole (BBT)^24^, borodipyrromethane (BODIPY)^25^, diketopyrrolopyrrole (DPP)^26^, and polyacrylonitrile frameworks^27^ etc. have been widely employed in NIR-II molecules. Through molecular engineering strategies such as dual receptor coupling, donor substitution, bridge unit regulation, and side chain modification, various NIR-II molecules have been developed, but their optical absorption is often limited to the NIR-IIa (1000-1500 nm) region. Therefore, there is an urgent need to design novel and more effective conjugated systems through original design to achieve PAI in the NIR-IIb region. Addressing these scientific hurdles could revolutionize clinical imaging capabilities, particularly for applications that require the suppression of endogenous background signals^28,29^, such as tumor margin delineation and intraoperative guidance, where current modalities face significant resolution constraints^30–32^. Thus, the development of NIR-IIb contrast agents is a crucial step for precision PAI diagnostics.

**Fig. 1:**
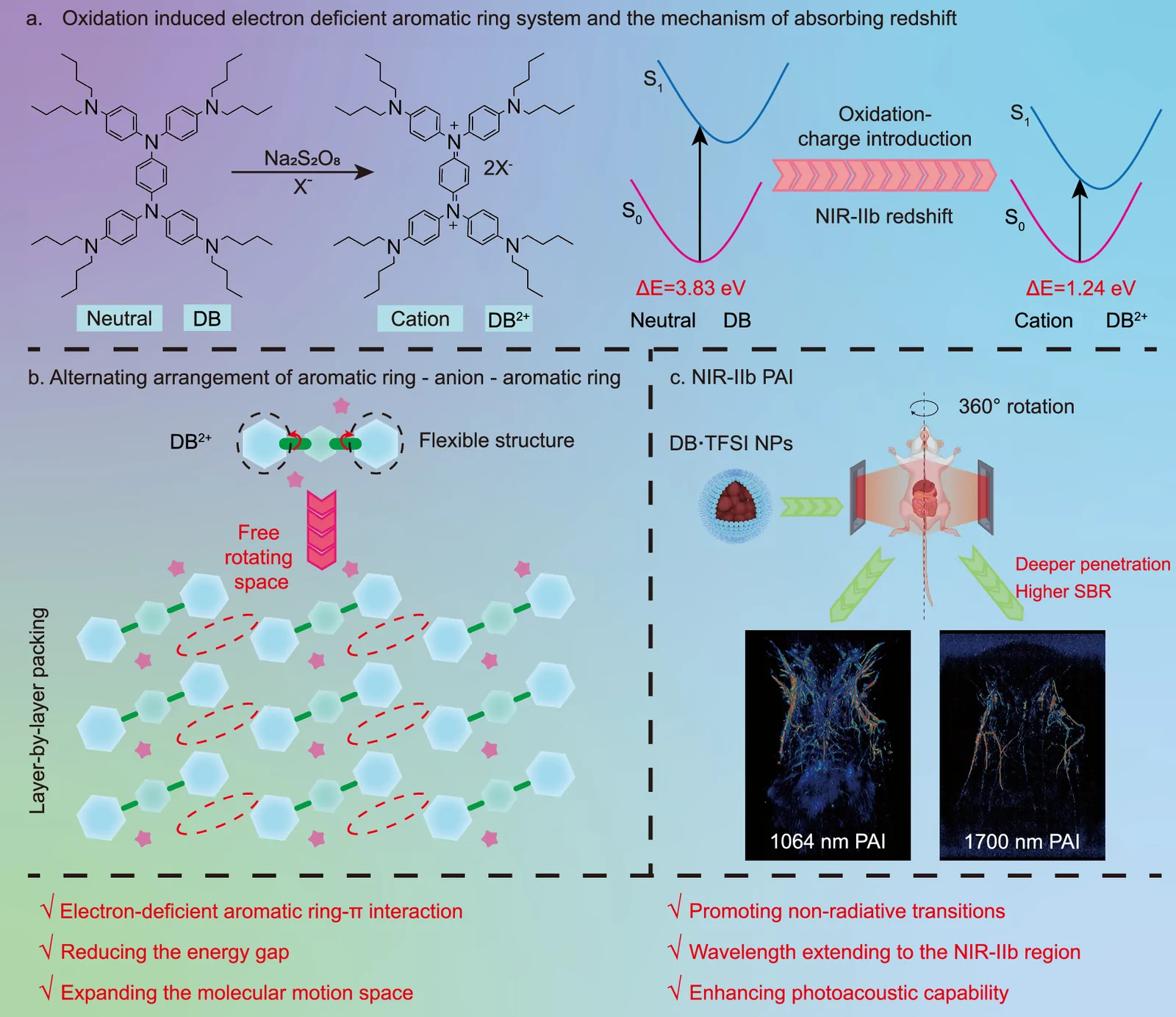
Design concept of DB·2X nano-agents for NIR-IIb PAI. **a** Oxidation induced electron deficient aromatic ring system and the mechanism of absorbing redshift. **b** Alternating arrangement of aromatic ring-anion-aromatic ring. **c** PAI under 1064 nm or 1700 nm excitation. Created in BioRender. Mou, X. (2026) https://BioRender.com/yenk1gz.

Herein, for the first time, we synthesized 1700 nm excitation PAI contrast dicationic nano-agents (named as DB·2X, X = bis(trifluoromethanesulfonyl)imide (TFSI^-^), borate oxalate (BOB^-^), or tetrafluoroborate (BF_4_^-^)) based on diimmonium (DB) molecules (Fig. 1). Spectroscopic and theoretical investigations revealed that anion-directed charge-transfer complexation induces significant electronic restructuring in the DB^2+^ cation. The highest occupied molecular orbital (HOMO) of neutral DB undergoes oxidative electron depletion, forming the lowest unoccupied molecular orbital (LUMO) of DB^2+^. Meanwhile, the HOMO-1 orbital in neutral DB is transformed into the HOMO orbital of DB^2+^. This electronic configuration enables symmetry-allowed localized excitation, generating enhanced NIR-II absorption characterized by intensified oscillator strength (f = 1.11) and reduced excitation energy (1.24 eV). Single-crystal X-ray diffraction analysis demonstrates extended intermolecular distances exceeding 5.0 Å in aggregated DB·2X, particularly DB·2TFSI exhibiting looser packing (~7.0 Å). This molecular architecture provides enhanced motional freedom for molecular dynamics, facilitating optimized non-radiative energy dissipation. Both in vitro and in vivo experiments demonstrated that the DB·2TFSI nano-agents exhibit exceptional PAI capabilities under 1700 nm pulsed laser excitation. Compared to conventional 1064 nm pulsed laser excitation, this system achieves improved contrast under the tested conditions, enhanced spatial resolution, and significantly improved SBR. This study not only establishes a molecular platform for 1700-nm excited PA nano-agents but also provides a robust theoretical and practical foundation for advancing future clinical translation of biomedical imaging technology.

## Results

### Molecular theoretical calculation of DB·2X

**Fig. 2:**
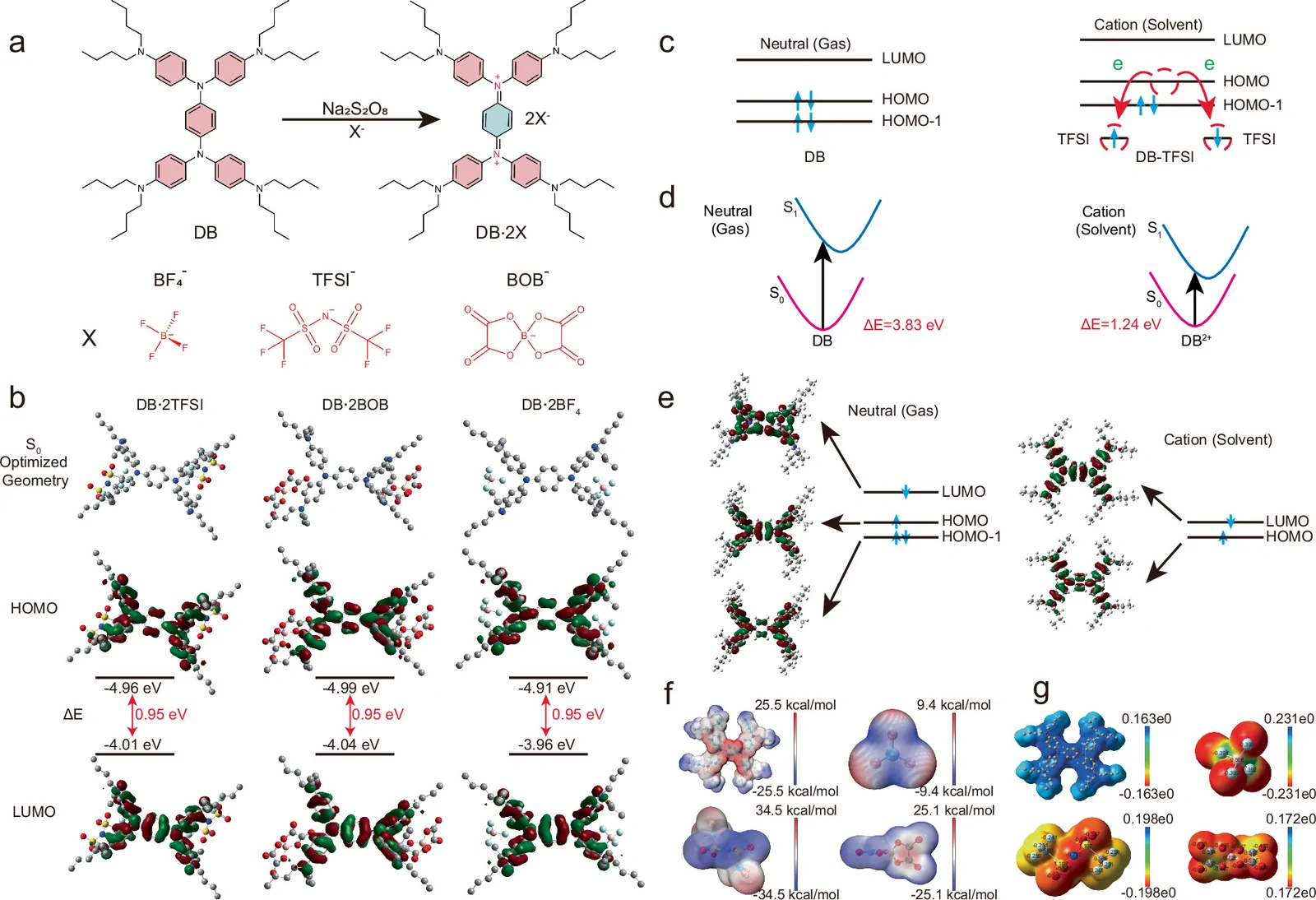
Molecular engineering and electronic structures of DB·2X. **a** Synthesis of DB·2X (X = BF_4_^-^, BOB^-^ and TFSI^-^). **b** The calculated HOMO, LUMO, and optimized S_0_ geometry of DB·2X. **c** The configurations of the first electronically excited state for the neutral molecule DB and the ionized DB^2+^. **d** The formation mechanism of ionized DB^2+^ in solution. **e** Comparison of vertical excitation energies between the neutral molecule DB and the ionized DB^2+^. **f** Surface ESP distribution map of DB and different ligand (BF_4_^-^, TFSI^-^, and BOB^-^). **g** Geometrically optimized structure, ESP and Mulliken charge distribution of DB and different ligand (BF_4_^-^, TFSI^-^ and BOB^-^).

DB molecule can coordinate with various salt compounds through a straightforward one-step reaction to obtain the DB·2X complexes (as illustrated in Fig. 2a and Supplementary Figs. 1, 2). To ascertain the optical absorption characteristics of DB·2X, we conducted calculations using density functional theory (DFT). Initially, we optimized the molecular geometries of these complexes in their ground state (S_0_), then optimized their HOMO and LUMO configurations within the S_0_ state (Fig. 2b). The results revealed that the HOMO and LUMO levels of all three DB·2X complexes are delocalized across the entire molecular framework. Notably, their energy gaps are uniformly 0.95 eV. This exceptionally small gap correlates with NIR-II absorption, likely attributed to extensive electron delocalization within the DB·2X framework. Following this, we theoretically computed the absorption spectra of these three molecules. As exhibited in Supplementary Fig. 3a–c, the absorption of all three molecules spans beyond 1000 nm, which is consistent with their energy gaps. When DB complexes with the ligand (e.g., TFSI^-^), electrons from neutral DB’s HOMO transfer to the ligand, forming the cationic DB (DB^2+^). After the charge transfer, the originally neutral DB’s HOMO loses electrons, and becomes the LUMO of DB^2+^, and the previously occupied orbital HOMO-1 of neutral DB transforms into the HOMO of DB^2+^ (Fig. 2c). Through theoretical calculations of the absorption spectrum, we found that for DB^2+^, its maximum absorption occurs during the transition from the S_0_ to the first electronically excited state (S_1_), with an excitation energy of 1.24 eV, which is less than the S_0_ → S_1_ excitation energy of 3.83 eV of neutral DB (Fig. 2d). This is the fundamental reason for the redshift in the spectrum. Specifically, the S_1_ state of DB^2+^ is primarily composed of the HOMO → LUMO excitation mode, which corresponds to the HOMO-1 → HOMO excitation in the originally neutral DB. The S_1_ state of neutral DB is also primarily composed of the HOMO → LUMO excitation mode (Fig. 2e). The HOMO-LUMO gap of DB^2+^ (0.94 eV) is significantly smaller than that of neutral DB (3.79 eV)^33^, consistent with the excitation energy results. Additionally, theoretically simulated absorption spectra indicate that the absorption spectrum of DB^2+^ (1002 nm) is in complete agreement with the absorption spectrum of DB·2TFSI (1150 nm) (Table 1). This suggests that the charge transfer mechanism is correct. From excited-state electronic structure calculations, we found that, compared to the neutral DB molecule, the ionic DB has a larger oscillator strength, implying a stronger absorption intensity. This is mainly attributed to their S_1_ excited electronic states being HOMO → LUMO excitation models, i.e., HOMO-1 → HOMO excitation in the neutral DB molecule. From the molecular orbitals, they are locally excited, thus possessing large absorption intensity. Meanwhile, we also conducted calculations of the surface electrostatic potential (ESP) for DB·2X complexes. As shown in Fig. 2f, for the DB parent molecule, the positive potential is mostly concentrated on the benzene rings, particularly with a very high positive potential on the central benzene ring. Subsequently, the positive potential gradually diminishes on the surrounding benzene rings connected to nitrogen, and as it extends towards the alkane chain, it becomes strongly negative. Furthermore, upon introducing anions, it is further observed that positive charges are predominantly centered on the main chain’s central nitrogen atom, with different anions being introduced onto the N^+^ of the main chain. As the calculated volume of the anions increases (BF_4_^-^ <BOB^-^ ≈ TFSI^-^), the number of electronegative atoms increases, which can influence the polarizability and the distribution of intramolecular charges. This allows for a better determination of which anion interacts more favorably with the DB cationic backbone. By examining the Mulliken charges of the counter anions, we further analyzed the degree of charge separation among different anions. The calculated Mulliken charges for various atoms within the anions are presented in Fig. 2g, of which the net charges of all three anions are −1. Subsequently, under the condition of identical net charges, as the volume of the anions gradually increases, the intramolecular charge distribution becomes more uniform. The presence of highly electronegative F atoms in BF_4_^-^ can produce a strong charge distribution, averaging the negative charges across the F atoms. In contrast, BOB^-^ and TFSI^-^ contain more electronegative atoms, such as O, N, and F. They all contribute to a more uniform distribution of negative charge throughout the molecule. Further observation of the anions’ ESP reveals that all three anions have clearly visible negative potential regions near F atoms, O atoms, and N atoms. The DB structure, especially the central benzene ring and N^+^ ions, possess positive potentials. Therefore, they are more prone to forming more stable complexes through anion-π^+^ interaction. Subsequently, the total interaction energy between DB and the anions was calculated. As shown in Supplementary Table 1, the three different interaction energy are 87.8 kcal/mol (BF_4_^-^), 104.4 kcal/mol (BOB^-^), and 108.0 kcal/mol (TFSI^-^). The total interaction energy aligns with the ESP results, indicating that the two anions, BOB^-^ and TFSI^-^, exhibit stronger anion-π^+^ interaction.

**Table 1:** Excitation energy, oscillator strength, electronic configuration, and HOMO-LUMO energy gap for the S_1_ state of DB, DB^2+^, DB·2BF_4_, DB·2TFSI, and DB·2BOB

Molecule	Excited Energy (eV)	Oscillator Strength (*f*)	HOMO-LUMO Gap (eV)
DB	3.83 (324 nm)	0.0229	3.79
DB^2+^	1.24 (1002 nm)	1.1146	0.943
DB·2BF_4_	1.08 (1150 nm)	1.2425	0.947
DB·2TFSI	1.08 (1150 nm)	1.2227	0.946
DB·2BOB	1.08 (1150 nm)	1.2036	0.945

### Single crystal analysis of DB·2X

**Fig. 3:**
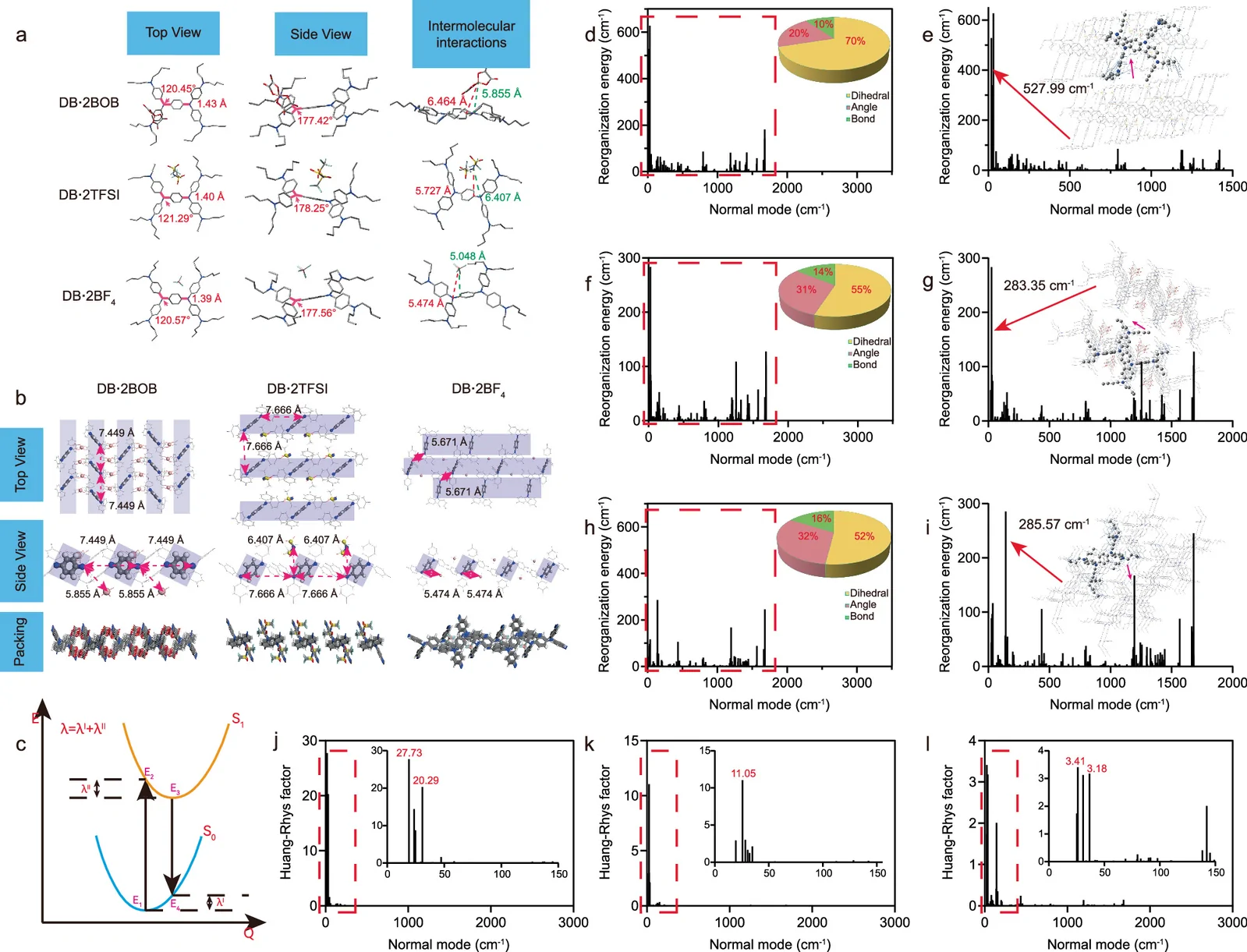
Molecular packing and non-radiative decay mechanisms of DB·2X. **a** Single-crystal structure of DB·2BOB, DB·2TFSI and DB·2BF_4_ in monomer mode (includes top view, side view and plots at intermolecular interactions). **b** Top view, side view and molecular packing of DB·2BOB, DB·2TFSI and DB·2BF_4_ in the aggregated state. **c** Schematic diagram of the adiabatic potential energy surface for four-point calculation. Calculated total recombination energy of **d** DB·2TFSI, **f** DB·2BOB, **h** DB·2BF_4_ at normal mode frequency and contribution of different vibration modes to the total recombination energy. In the crystalline phase, the recombination energy of **e** DB·2TFSI, **g** DB·2BOB, and **i** DB·2BF_4_ with representative vibrational modes varies with the frequency of the forward mode. **j**–**l** Calculated HR factors of DB·2TFSI, DB·2 BOB, and DB·2BF_4_ at normal mode frequency.

Based on theoretical calculations, we further investigated the molecular conformation and packing in the single-crystal state. Initially, Single crystals of the three target molecules (DB·2BOB, DB·2TFSI, and DB·2BF_4_) were successfully grown via low-temperature evaporation from dichloromethane solution. The crystal data and X-ray crystallographic acquisition parameters are summarized in Supplementary Table 2 and Fig. 3a depicts the monomeric structure of the three molecules, which exhibit spatially distorted molecular structures that are generally consistent with the optimized structure obtained from the theoretical calculation. The parent structures of the three molecules exhibit relatively twisted bond angles that are not significantly influenced by the anions. Upon introducing different anions, it was observed that in DB·2BOB and DB·2TFSI, the distances from the central anions (B atom and N atom) to the central phenyl ring are 6.464 Å and 5.727 Å, respectively. Additionally, the distances from the central anions (B atom and N atom) to the central N atom are 6.464 Å (B···N) and 6.407 Å (N···N), respectively. However, in DB·2BF_4_, the distance from the central anion (B atom) to the central phenyl ring is 5.048 Å, and the distance from the central anion (B atom) to the central N atom is 5.474 Å (B···N). Therefore, it is reasonable to infer that these three molecules can effectively prevent intermolecular π-π stacking. Due to the larger steric hindrance of the BOB^-^ and TFSI^-^ anions, the weak interaction distances between the parent structure and ligands in DB·2BOB and DB·2TFSI are much greater than in DB·2BF_4_. Consequently, in the monomeric state, DB·2BOB and DB·2TFSI exhibit better intramolecular mobility. Subsequently, we conducted various analyses of the molecular packing morphology of polymers and different molecules. The results shown in Fig. 3b, indicating that the π-π interaction distances between adjacent phenyl rings in DB·2BOB and DB·2TFSI are 7.449 Å and 7.666 Å, respectively, which are significantly larger than the π-π interaction distance between adjacent phenyl rings in DB·2BF_4_ (5.671 Å). Therefore, the presence of anions provides ultra-long intermolecular interaction distances, and as the anion volume increases, greater steric hindrance emerges, further demonstrating that in the molecular packing state, molecules can move better without interfering with each other, reducing intermolecular interaction. Consequently, DB·2BOB and DB·2TFSI demonstrate superior intramolecular mobility in the monomeric state, while their extended intermolecular distances minimize electronic coupling in the solid state. These structural insights provide strong evidence that anion engineering can effectively regulate the non-radiative decay pathway of the NIR-II DB·2X system. The ultralong intermolecular distance achieved through spatial shielding not only reduces intermolecular interactions, but also establishes a design paradigm for optimizing the photophysical properties of π-conjugated materials.

### Non-radiative decay processes of DB·2X

During the electronic transition, there are two pathways for energy to return from the S_1_ state to the S_0_ state: radiative transition and non-radiative transition^33–36^. Preliminary investigations based on the aforementioned results indicate that the presence of anions leads to different steric hindrances among the three DB·2X molecules, isolating the parent molecules from each other. Therefore, we reasonably suspect that these three molecules can effectively undergo intramolecular motion. To further explore the intramolecular motion and understand which type of motion dominates, we introduce the concepts of reorganization energy (λ) and Huang-Rhys (HR) factor (Eqs. 1–6)^37,38^. Through the four-point calculation on the schematic diagram of the adiabatic potential energy surface, it can be concluded that:

1 $$\lambda={\lambda }^{I}+{\lambda }^{II}\ldots \ldots \ldots$$

2 $${\lambda }^{I}={{{\rm{E}}}}_{4}-{{{\rm{E}}}}_{1}\ldots \ldots \ldots$$

3 $${\lambda }^{II}={{{\rm{E}}}}_{2}-{{{\rm{E}}}}_{3}\ldots \ldots \ldots$$

Among them, E_1_ represents the minimum energy in the S_0_ state, E_2_ is the total energy of the S_1_ state at the equilibrium geometry of the S_0_ state, E_3_ is the minimum energy in the S_1_ state, and E_4_ is the total energy of the S_0_ state at the equilibrium geometry of the S_1_ state. Meanwhile, λ can also be expressed as the sum of the contributions from normal mode (NM) relaxation in the harmonic oscillator approximation.

4 $${\lambda }^{{{\rm{I}}}}=\sum {{\rm{k}}}\epsilon {{\rm{I}}}\,{\lambda }_{{{\rm{k}}}}=\sum {{\rm{k}}}\epsilon {{\rm{I}}}\,\hslash {{{\rm{\omega }}}}_{{{\rm{k}}}}\,{{{\rm{HR}}}}_{{{\rm{k}}}}\ldots \ldots \ldots$$

5 $${\lambda }^{{{\rm{II}}}}=\sum {{\rm{k}}}\epsilon {{\rm{II}}}\,{\lambda }_{{{\rm{k}}}}=\sum {{\rm{k}}}\epsilon {{\rm{II}}}\,\hslash {{{\rm{\omega }}}}_{{{\rm{k}}}}\,{{{\rm{HR}}}}_{{{\rm{k}}}}\ldots \ldots \ldots$$

6 $${{{\rm{HR}}}}_{{{\rm{K}}}}={{{\rm{\omega }}}}_{{{\rm{k}}}}{{{\rm{D}}}}_{{{\rm{K}}}}^{2}/2\hslash \ldots \ldots \ldots$$

HR_k_ represents the HR factor for the kth mode. D_k_ denotes the displacement of the kth mode between the equilibrium geometry of the S_0_ state and S_1_ state. ω_k_ represents the vibrational frequency of the kth mode. These are used to assess intrinsic geometric changes between the S_1_ and S_0_ states in the single-crystal state, which play a crucial role in the non-radiative decay process. Consequently, to further analyze the motion patterns of the three molecules and the influence of λ on non-radiative decay, we employed Dushin software^39^ to assess the impact of vibrational frequency data on the total λ of the geometrically optimized structure. Initially, we performed geometric optimization on the molecules in the S_0_ state (Supplementary Fig. 4a–c). Subsequently, we calculated the total λ for DB·2BOB, DB·2TFSI, and DB·2BF_4_ to be 2321.93 cm^-1^, 4812.97 cm^-1^, and 3038.88 cm^-1^, respectively (as shown in Table 2). Through the calculation based on four points (Fig. 3c), we can obtain the total λ. Among the energy contribution of the three molecules, we found that changes in dihedral angles contribute the most to the total λ (as shown in Fig. 3d, 3f and 3h), with contributions of 3378.22 cm^-1^ (70% for DB·2TFSI), 1284.57 cm^-1^ (55% for DB·2BOB and 1571.08 cm^-1^ (52% for DB·2BF_4_), respectively. Changes in dihedral angles belong to the molecular motion mode in the low-frequency region, which significantly accelerates non-radiative decay, rapidly dissipating S_1_ state energy as heat. To further analyze the influence of vibration modes on reorganization energy, we observed that the primary contribution of frequency modes to reorganization energy are distributed between 0-1500 cm^-1^. Within this range, the modes with the largest contribution values for the three molecules are 527.99 cm^-1^ and 627.18 cm^-1^ (for DB·2TFSI, Fig. 3e and Supplementary Fig. 5d), 283.35 cm^-1^ and 127.56 cm^-1^ (for DB·2BOB, Fig. 3g and Supplementary Fig. 5e), and 285.57 cm^-1^ and 245.30 cm^-1^ (for DB·2BF_4_, Fig. 3i and Supplementary Fig. 5f). The results indicate that the reorganization energy of the DB·2X system is primarily contributed by low-frequency vibration, and these vibration modes may play a decisive role in the photothermal effect. Meanwhile, we have introduced the relationship between the HR factor and frequency modes. By projecting the energy distribution of the reorganization energy onto the internal coordinates, the corresponding results are shown in Fig. 3j–l, where the maximum values of the HR factor for all three molecules are concentrated below 50 cm^-1^. This indicates that low-frequency modes have a significant impact on the contribution to the reorganization energy.

**Table 2:** Total reorganization energy and the energetic contributions of bond lengths, bond angles, and dihedral angles to the total reorganization energy

	Dihedral (cm^−1^)	Bond angle (cm^−1^)	Bond length (cm^−1^)	Total (cm^−1^)
DB·2TFSI	3378.22	947.51	487.24	4812.97
DB·2BOB	1284.57	723.67	313.69	2321.93
DB·2BF_4_	1571.08	982.4	485.4	3038.88

### Morphological characterization of DB·2X nano-agents

**Fig. 4:**
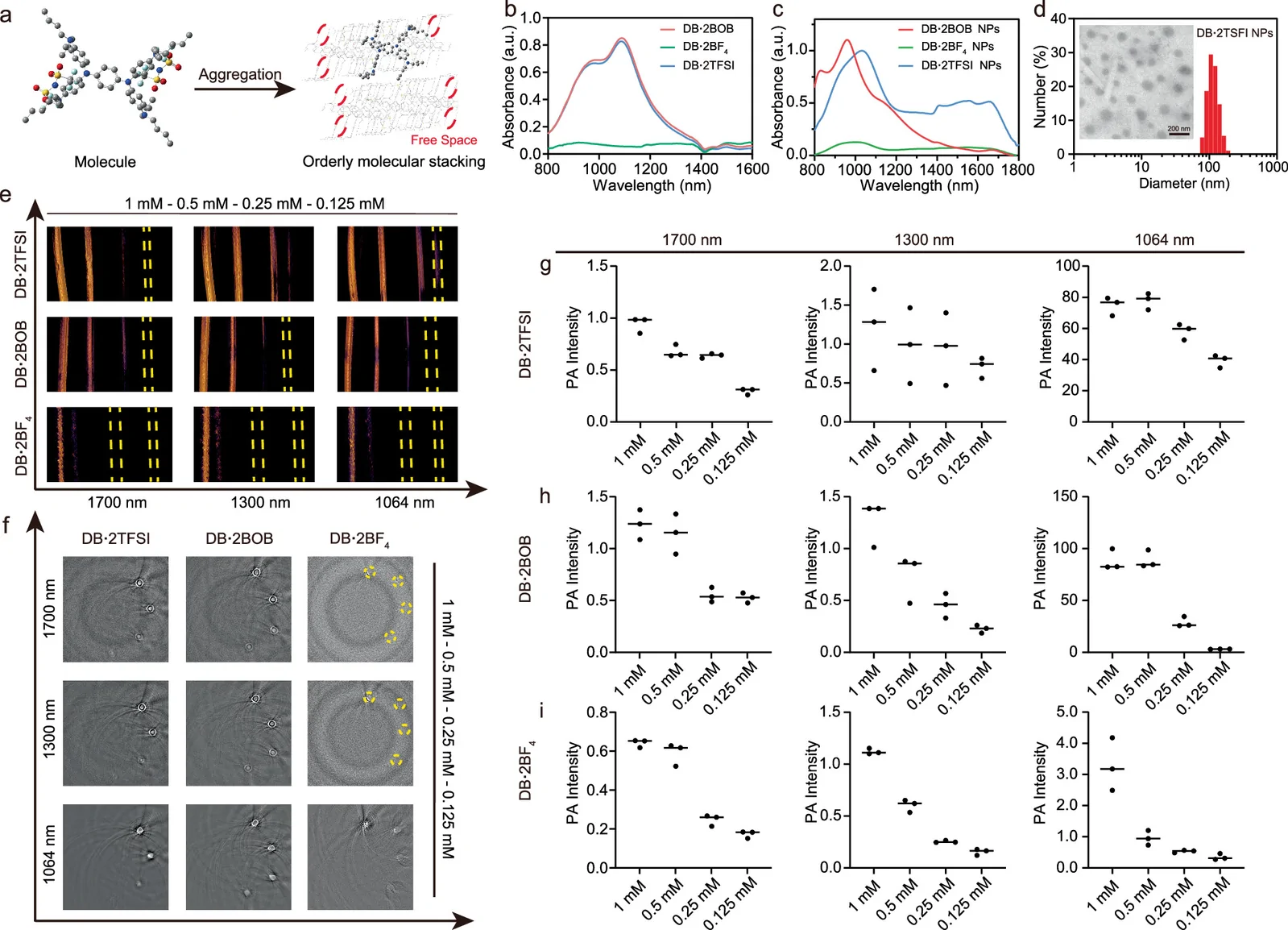
Characterization and in vitro photoacoustic performance of DB·2X NPs. **a** Schematic illustration of the fabrication process of DB·2X NPs. **b** The UV absorption spectra of different DB·2X in THF. **c** The UV absorption spectra of different DB·2X NPs in D_2_O. **d** TEM and DLS of DB·2TFSI. **e** PA signal of different NPs at various concentrations and under excitation by different pulsed laser source (1064 nm, 1300 nm, and 1700 nm). **f** Cross-sectional signal plots derived from (**e**). **g** PA signal of DB·2TFSI NPs at different concentrations (pulsed laser source: 1700 nm, 1300 nm, and 1064 nm, *n* = 3). **h** PA signal of DB·2BOB NPs at different concentrations (pulsed laser source: 1700 nm, 1300 nm, and 1064 nm, *n* = 3). **i** PA signal of DB·2BF_4_ NPs at different concentrations (pulsed laser source: 1700 nm, 1300 nm, and 1064 nm, *n* = 3). (The experiments of Fig. 4d–f were independently repeated three times with similar results, and one representative result is shown).

As shown in Fig. 4a, through nanoprecipitation, disordered molecules can aggregate into orderly stacked nanoparticles (NPs). Moreover, there is a certain amount of free space between molecules for molecular motion, effectively inhibiting radiative transition. NPs modified with PEG can significantly enhance their dispersion and stability in aqueous solution. Subsequently, we tested the absorbance spectra with three DB·2X complexes. The results revealed that all three molecules exhibited good NIR-II absorption capacity in 750–1600 nm range, and displayed very strong absorption peaks at 1100 nm as well as good absorption at 1600 nm (Fig. 4b). The experimental results in Fig. 4c show that after preparing these DB·2X complexes into NPs, good NIR-IIb absorption beyond 1700 nm was observed. As illustrated in Fig. 4d and Supplementary Fig. 6, we measured the particle sizes of three types of NPs using transmission electron microscopy (TEM) and dynamic light scattering (DLS): 100 nm for DB·2TFSI NPs, 40 nm for DB·2BOB NPs, and 200 nm for DB·2BF_4_ NPs. And the DB·2X NPs exhibit excellent long-term stability in aqueous solution without any degradation (Supplementary Fig. 7). In addition, all three types of DB·2X NPs exhibit negative Zeta potential, which is beneficial for maintaining colloidal stability in physiological environments and avoiding non-specific aggregation in vivo (Supplementary Fig. 8). Based on the analysis of the above experimental results, it is concluded that most of the energy of these three molecules returns from the S_1_ state to the S_0_ state through non-radiative decay pathway. To quantify the photothermal performance, we employed infrared imaging technology to measure the temperature changes of these three types of NPs under 1064 nm laser and 1550 nm laser irradiation. Firstly, we conducted heating tests on these three types of NPs at different concentrations. Since pure water exhibits strong signal interference at 1550 nm, which can affect the heating of the NPs themselves, we replaced the solution of NPs irradiated by the 1550 nm laser with deuterium oxide to eliminate background interference. As shown in Supplementary Fig. 9a and 9 d, after irradiation with 1064 nm laser and 1550 nm laser, the temperature of DB·2TFSI NPs increased steadily, reaching above 50 °C when the concentration increased to 200 μg/mL. Meanwhile, after laser irradiation (1064 nm and 1550 nm), the temperature of DB·2BOB NPs and DB·2BF_4_ NPs could also exceed 50 °C (Supplementary Fig. 9b, 9c, 9e and 9 f). Subsequently, we adjusted the power of the laser. As shown in Supplementary Fig. 10, these results indicate that all three types of NPs exhibited temperature increases with increasing laser power. Additionally, these three types of NPs maintained temperature above 50 °C without attenuation during five heating/cooling cycles (Supplementary Fig. 11). Therefore, DB·2X NPs exhibit excellent photothermal performance, laying a solid foundation for subsequent in vitro and in vivo PAI.

As shown in Supplementary Fig. 12, we conducted in vitro PAI analysis of three DB·2X NPs using three different pulsed laser sources. Firstly, we selected a 1064 nm pulsed laser source for in vitro PAI of DB·2X NPs that all three NPs exhibited effective PAI performance at 1064 nm (Supplementary Fig. 12b). Subsequently, we chose a 1300 nm pulsed laser source for imaging. The results shown in Supplementary Fig. 12c indicated that the PA signal intensities of all three NPs decreased significantly, especially for DB·2BF_4_, which had a PA signal intensity of approximately 0.5. As shown in Supplementary Fig. 12a, after excitation with the 1700 nm pulsed laser source, DB·2TFSI NPs and DB·2BOB NPs still exhibited bright signals, indicating their excellent deep PAI capability. However, DB·2BF_4_ NPs did not produce a strong PA signal under excitation with the 1700 nm pulsed laser source (Supplementary Fig. 12d). Next, we performed PAI of the three NPs at different concentrations. The results shown in Fig. 4e–i revealed that as the solution concentration increased, all three NPs exhibited a trend of increasing PA signal intensity. At a concentration of 0.125 mM, DB·2TFSI still had a relatively strong PA signal. Regardless of whether excited by the 1700 nm, 1064 nm, or 1300 nm pulsed laser sources, DB·2BF_4_ NPs exhibited weak PA signal intensity at concentrations of 0.5 mM or lower. Therefore, combining previous theoretical calculations and single-crystal data, we believe that the coordination of TFSI^-^ in DB·2TFSI molecules results in greater steric hindrance, facilitating molecular motion and enabling energy to return to the S_0_ state more efficiently through non-radiative transition. This ultimately produces stronger acoustic signals and presents high-contrast in vitro PAI.

### In vivo PAI

**Fig. 5:**
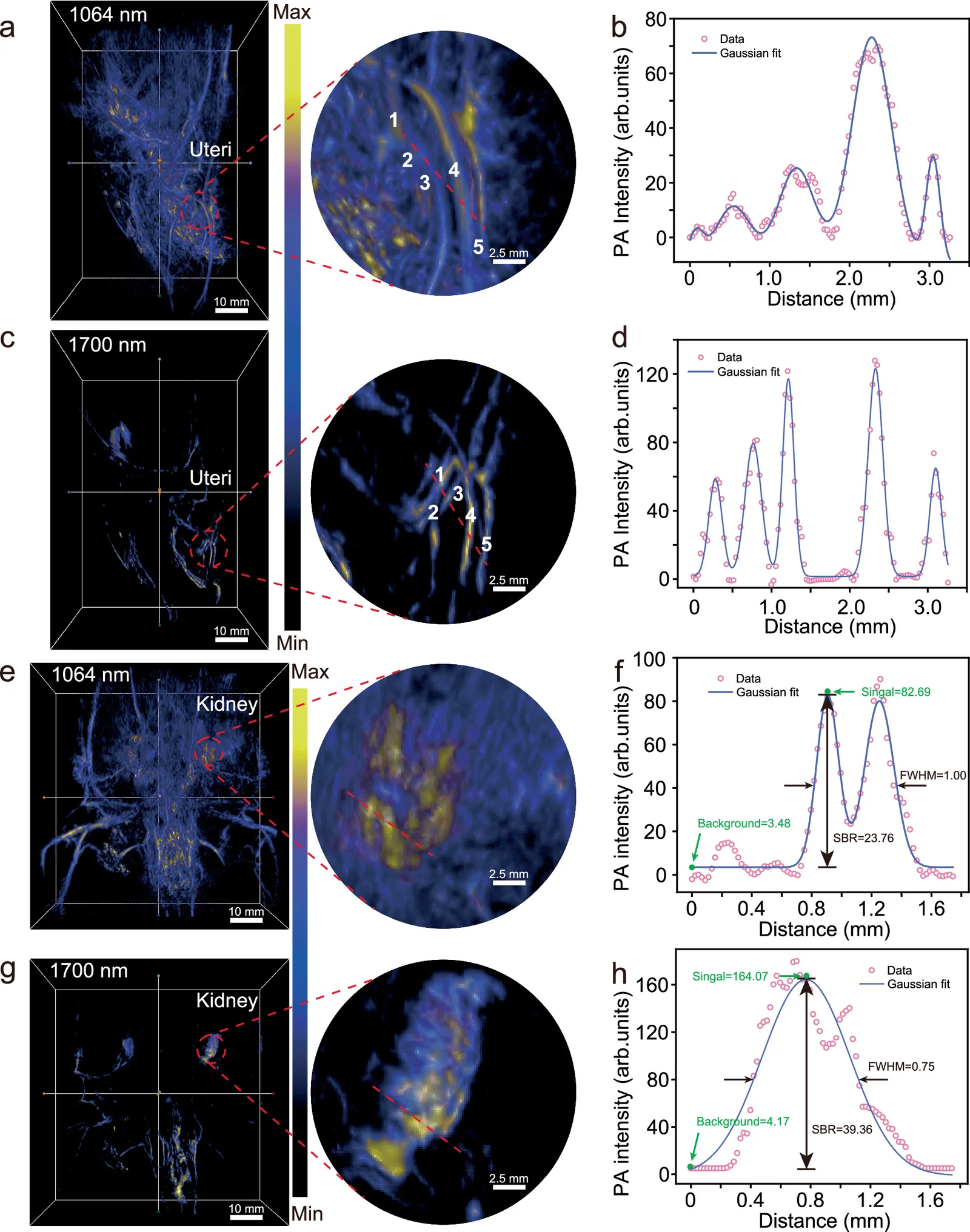
In vivo NIR-IIb PAI in the uterine adhesion model. **a** PAI images excited by the 1064 nm pulsed laser at the uterine site (*n* = 3). **b** PA signal intensity of the blood vessel marked by a red line from (**a**). **c** PAI images excited by the 1700 nm pulsed laser at the uterine site (*n* = 3). **d** PA signal intensity of the blood vessel marked by a red line from (**c**). **e** PAI images excited by the 1064 nm pulsed laser at the kidney site (*n* = 3). **f** PA signal intensity of the blood vessel marked by a red line from (**e**). **g** PAI images excited by the 1700 nm pulsed laser at the kidney site (*n* = 3). **h** PA signal intensity of the blood vessel marked by a red line from (**g**). (The experiments of Fig. 5a, c, e, g were independently repeated three times with similar results, and one representative result is shown).

Before conducting biological experiments, we first verified the biological toxicity of DB·2X. We first selected two normal cell lines: HUVEC cells (human umbilical vein endothelial cells) and L929 cells (mouse fibroblasts) to conduct toxicity tests. As shown in Supplementary Fig. 13a, b, different DB·2X NPs were added to these normal cell lines. The results indicated that the NPs exhibited minimal cytotoxicity towards normal cells, to the extent that they did not even affect the proliferation of these cells. Subsequently, we chose DB·2TFSI with good PAI performance for in vivo experiment. Prior to conducting the imaging experiment, we further tested the toxicity of DB·2TFSI NPs in vivo. Normal SD rats aged between 12–14 weeks were selected, and DB·2TFSI NPs were injected via the tail vein. After allowing for in vivo circulation, the rat was euthanized, and its heart, liver, spleen, lung, and kidney were harvested for hematoxylin and eosin (HE) staining and sectioning. Compared to the sections of the control group (SD rats that did not receive NPs injection), DB·2TFSI NPs also did not exhibit any toxic effects on the organisms (as shown in Supplementary Fig. 14). To verify the preliminary safety biocompatibility of the DB·2TFSI NPs in this study, we conducted a short-term toxicity assay via tail vein injection in mice, and detected and analyzed the blood routine, liver function and renal function indices on days 7, 14 and 21, respectively. The results showed that all the above indices of all mice remained within the normal ranges (Supplementary Table 3 and Supplementary Table 4). To test PAI performance in deep tissues, we used a mouse model of uterine adhesion^40^ to observe the imaging effects of both lesion sites and kidney sites. We selected normal female mice aged 8-10 weeks to establish a disease model in the uterine area (as shown in Supplementary Fig. 15a-b). Subsequently, we administered DB·2TFSI NPs (1 mM, 200 μL) vaginally to the mice for PAI. As shown in Figs. 5a, 5c, and Supplementary Movies 1 and 2. We performed PAI on mice with uterine cavity adhesion using pulsed laser source at 1700 nm, and 1064 nm for comparison. The results revealed that the optical resolution and contrast obtained with the 1700 nm pulsed laser source were clearer than those obtained with the 1064 nm pulsed laser source, demonstrating the excellent PAI performance of DB·2TFSI NPs in the NIR-IIb region. The images excited by the 1064 nm pulsed laser source exhibited lower contrast due to the large amount of water and hemoglobin in mice, which can absorb photons excited by the 1064 nm laser source, resulting in interference signals in the background and subsequently making the image contrast more blurred. In contrast, photons excited by the 1700nm pulsed laser source resulted in better contrast and lower self-interference signal in the uterus and surrounding blood vessels. Furthermore, 3D NIR-II PAI enabled detailed visualization of vascular architecture in the uterine region. Five peri uterine vessels (labeled 1-5 from left to right) were magnified and analyzed using ImageJ for signal intensity quantification. As demonstrated in Fig. 5b and 5d, the 1700 nm pulsed laser excitation yielded signal intensities of 58.51, 79.24, 117.2, 123.04, and 64.72 for vessels 1-5 respectively, representing 14.96-fold (vs 3.91), 6.95-fold (vs 11.40), 4.62-fold (vs 25.37), 1.68-fold (vs 73.12), and 2.19-fold (vs 29.55) enhancements compared to 1064 nm pulsed laser excitation. Gaussian fitting revealed vessel widths of 0.44 mm (1), 0.52 mm (2), 0.46 mm (3), 0.64 mm (4), and 0.39 mm (5) under 1700 nm excitation, versus 0.23 mm (1), 0.68 mm (2), 0.61 mm (3), 1.12 mm (4), and 0.41 mm (5) respectively for 1064 nm excitation. These findings collectively demonstrate that DB·2TFSI NPs under 1700 nm excitation significantly reduce background interference, enabling high-resolution imaging with suppressed background signals. To further validate the reduced self-interference of DB·2TFSI NPs under 1700 nm excitation, we analyzed peripheral signal intensity in renal imaging. Figure 5e, 5g and Supplementary Movies 1 and 2 reveal superior contrast and anatomical delineation in 1700 nm-excited renal images compared to 1064 nm counterparts. Quantitative analysis of a selected renal region (demarcated by red dashed lines) showed a SBR of 39.36 with a full-width at half-maximum (FWHM) of 0.75 for 1700 nm excitation (Fig. 5h), significantly outperforming 1064 nm excitation, which exhibited an SBR of 23.76 and a broader FWHM of 1.00 (Fig. 5f). This enhanced contrast reaffirms the great performance of 1700 nm pulsed laser excitation in minimizing background interference while maintaining signal fidelity.

**Fig. 6:**
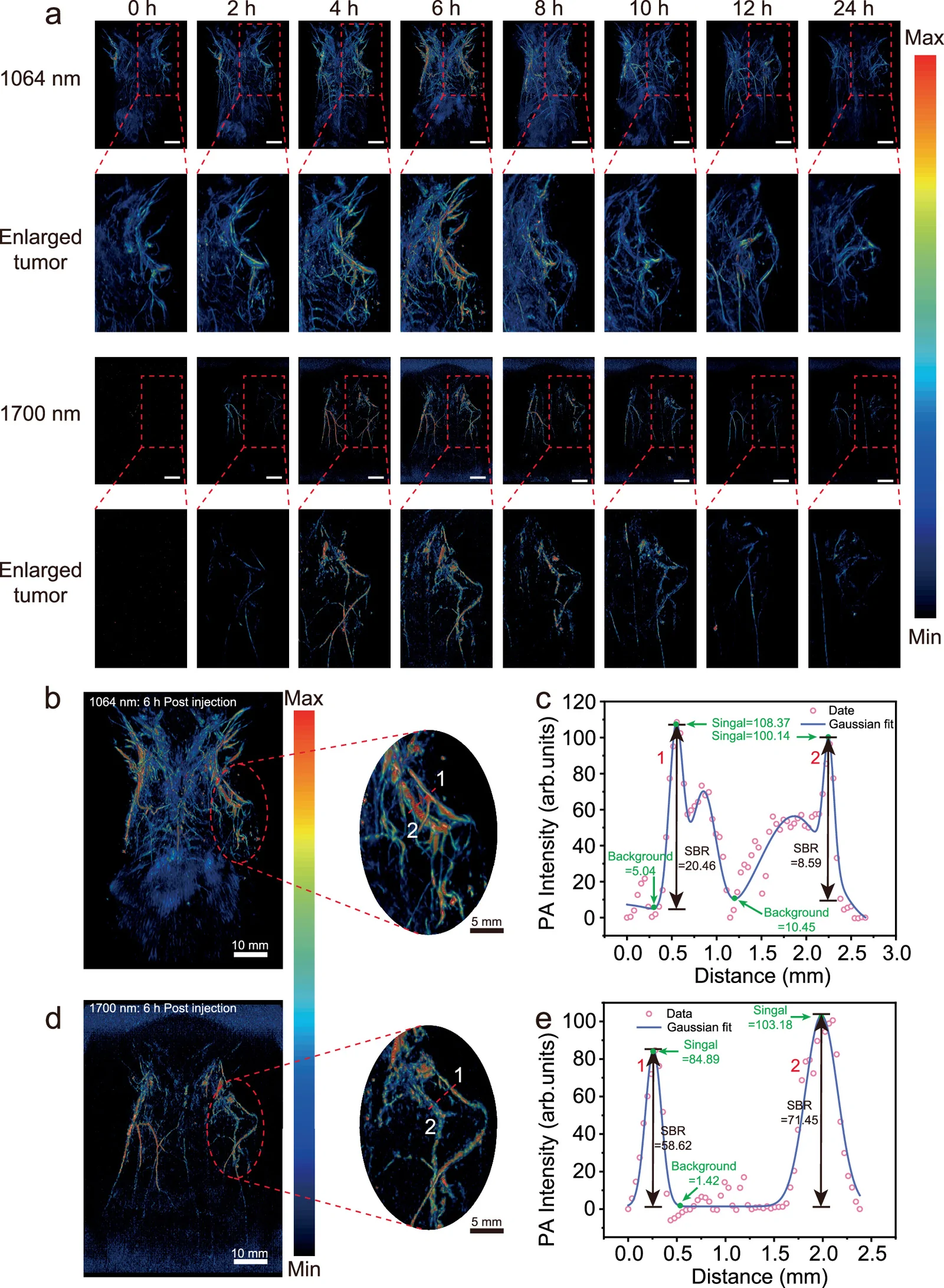
In vivo NIR-IIb PAI of subcutaneous tumors. **a** In vivo PAI and tumor local magnification of subcutaneous tumor sites in mice (*n* = 3) using different laser sources at different time points. Scale bar: 10 mm. PAI stimulated by 1064 nm (**b**) or 1700 nm (**d**) pulsed laser 6 hours after intravenous injection of DB·2TFSI NPs, and the red line annotation of the interested blood vessels in the local magnified image of the tumor. Gaussian fitting curve of PA intensity versus distance for PAI excited by 1064 nm (**c**) or 1700 nm (**e**) pulsed light source. (The experiments of Fig. 6a, b, d were independently repeated three times with similar results, and one representative result is shown).

We then conducted systematic in vivo studies using a subcutaneous tumor mouse model to evaluate the NIR-II PAI performance of DB·2TFSI NPs in tumor tissues. Following intravenous administration of 1 mM DB·2TFSI NPs, longitudinal PAI was performed to quantitatively assess time-dependent changes in PA signal and contrast enhancement. As depicted in Fig. 6a, at the 0-hour time point, in contrast to PAI excited by the 1700 nm pulsed laser source, the PAI triggered by the 1064 nm pulsed laser source presents a relatively distinct imaging contour. Owing to the high content of hemoglobin in blood vessels, which possesses a certain absorption capacity for the 1064 nm wavelength laser, when the laser from the 1064 nm pulsed source irradiates the vascular region, hemoglobin can efficiently absorb light energy. This energy absorption is subsequently converted into thermal energy, leading to an instantaneous rise in the temperature of the local tissue. The rapid heating induces thermal expansion of the tissue, generating pressure waves that are transduced into PA signal. Notably, within tumor tissues and their adjacent normal tissues, where active angiogenesis occurs, strong PA signal can also be elicited. Additionally, at the 0-hour mark, bones exhibit a degree of light absorption for the 1064 nm pulsed laser source. Following light energy absorption, a minor thermal expansion ensues, thereby generating PA signal. In contrast, PAI excited by the 1700 nm pulsed laser source fails to produce discernible PAI at the 0-hour time point. The underlying reason lies in the fact that 1700 nm falls within the NIR-IIb window of the light source. Within this window, biological tissues exhibit strong light scattering and weak absorption at these wavelengths, precluding the accumulation of sufficient energy to induce significant thermal expansion. Consequently, the pressure waves are scarcely detectable by the detector, ultimately resulting in no apparent PA signal. After administration, the PA signal of DB·2TFSI NPs in the tumor area increased over time. After 10 hours, the PA signal of DB·2TFSI NPs gradually weakened near the tumor area. To further underscore the advantages of the 1700 nm PA signal, the PA signal graphs of 1064 nm pulsed laser and 1700 nm pulsed laser at 6 hours post-tail vein injection were selected for analytical comparison. As illustrated in Fig. 6b, d and Supplementary Movies 3 and 4, the PA signal of the 1700 nm pulsed laser demonstrates reduced interference from in vivo background signal, furnishing clearer visualization regarding the morphology of selected tumor-associated vascular features. Conversely, the PA signal image from the 1064nm pulsed laser is affected by partial vascular background interference, making it difficult to distinguish normal from abnormal blood vessels within the tumor tissue. Furthermore, two blood vessels of interest within the tumor were demarcated with red dashed lines, and Gaussian fitting analysis was performed on their respective structures. As shown in Fig. 6c and 6e, the signal intensity of blood vessels 1-2 excited by the 1700 nm pulsed laser are 84.89 and 103.18, respectively. For the 1064 nm pulsed laser, the signal intensity for blood vessels 1-2 are 108.37 and 100.14, respectively. Although the signal intensity excited by the 1700 nm pulsed laser is marginally lower, it is accompanied by an extremely low background signal (background signal intensity: 1.42). Moreover, the SBR for the two blood vessels excited by the 1700 nm pulsed laser were calculated to be 58.62 and 71.45, respectively. Compared with the 1064 nm pulsed laser excitation approach, enhancements of 2.87-fold (vs 20.46) and 8.32-fold (vs 8.59) were achieved, respectively. This indicates that 1700 nm exhibits superior background suppression for weak signals and fine structures in the tumor area, facilitating clearer identification of micro-vessels and tumor boundaries. In summary, under the tested imaging condition, 1700 nm excitation reduced endogenous background and improved signal-to-background contrast compared with 1064 nm excitation, facilitating clearer visualization of selected tumor-associated vascular and structural features. These findings support its potential utility for contrast-enhanced tumor PAI.

## Discussion

This study reports the development of DB·2X nano-agents optimized for 1700 nm excitation, expanding the available molecular platforms for NIR-IIb PAI. By leveraging the unique electronic and structural properties of DB·2X, we achieved broad NIR-II absorption extending toward 1700 nm, with enhanced oscillator strength and enhanced non-radiative energy dissipation, thereby supporting improved PA contrast in vivo imaging. DFT calculations reveal that DB^2+^ exhibits a dramatically reduced excitation energy and enhanced oscillator strength by molecular engineering, attributed to a symmetry-allowed local excitation mechanism for electronic restructuring. Single-crystal X-ray diffraction revealed loose molecular packing and enlarged intermolecular separations, which are consistent with increased conformational freedom and promoted non-radiative energy dissipation. The loose molecular packing and non-radiative decay pathways of DB·2X nano-agents amplify PA signals, while their good biocompatibility and low cytotoxicity underscore their clinical potential. Compared to 1064 nm excitation, DB·2TFSI NPs under 1700 nm excitation improved signal-to-background contrast and clearer delineation of selected vascular features, sharper anatomical delineation, and improved SBR in uterine adhesion and tumor models. These improvements stem from the NIR-IIb window’s reduced photon scattering and reduced interference from endogenous chromophores. By bridging the gap between preclinical innovation and clinical application, DB·2X nano-agents could provide a molecular basis for further investigation of 1700 nm-excitable organic PA contrast agents.

## Methods

### Materials

All chemical reagents used in the experiments were purchased from Adamas. DSPE-PEG_2000_-COOH was acquired from Shanghai Yare Biotech, Inc. and CCK-8 was sourced from TCI Huacheng Industrial Development Co., Ltd (Shanghai, China). Fetal bovine serum was purchased from Biological Industries Israel Beit Haemek LTD. DMEM medium was obtained from BI Shanghai Co., Ltd. The solvents used were all deionized water and phosphate-buffered saline. The UV-NIR absorption spectra of the compounds were recorded using a UV-3600 Plus spectrophotometer. The relative molecular weight of the compound was determined by Thermo Fisher Scientific Orbitrap Exploris 120. The FT-IR spectra of the compound were detected using a Thermo Fisher Scientific Nicolet iS20 instrument. The particle size distribution of the NPs was evaluated using a Zetasizer nano ZS particle analyzer (Malvern Instruments Ltd.). TEM images were recorded with a JEM 2100 F microscope. Single-crystal X-ray diffraction (SXRD) analysis was conducted using a Bruker Smart Apex with a molybdenum target at room temperature, collecting single crystals of different compounds at a scan rate of 5° min⁻¹. The crystal structures were solved using Olex2 software. In vitro and in vivo PAI were collected by TOMOWAVE (USA).

### Synthesis of DB·2TFSI

In a 50 mL single-neck flask, 0.90 g of N,N,N’,N’-tetrakis[4-(dibutylamino)phenyl]-p-phenylenediamine (0.97 mmol), 0.56 g of lithium bis(trifluoromethanesulfonyl)imide (1.94 mmol), 0.30 g of Na_2_S_2_O_8_ (1.26 mmol), and 20 mL of acetone were added. The reaction mixture was stirred at 60 °C for 1 hour under dark condition. After cooling the reaction mixture to room temperature, 20 mL of acetone was added for washing, followed by evaporation and drying of the solvent. The dried material was dissolved in 10 mL of CH_2_Cl_2_, and then a large amount of water was added for extraction. The organic phase containing the solution was retained and placed in a vacuum oven at 40 °C for evaporation and drying. Subsequently, a small amount of dichloromethane was added to the dried material for dissolution. A large amount of n-hexane was then added for recrystallization, and the recrystallization process was repeated three times. The final product, DB·2TFSI, was obtained for the subsequent experiment. Total ion chromatogram in the positive ESI mode of the DB·2TFSI. m/z calcd for C_62_H_92_N_6_^+^ [M]^+^: 920.7378; found, 920.7432; calcd for C_62_H_92_N_6_^+^ [M]^2+^: 460.3686; found, 460.3710.

### Synthesis of DB·2BOB

In a 50 mL single-neck flask, 0.50 g of N,N,N’,N’-tetrakis[4-(dibutylamino)phenyl]-p-phenylenediamine (0.54 mmol), 0.25 g of lithium bis(oxalato)borate (1.29 mmol), 5 mL of CH_2_Cl_2_, and 2 mL of ethanol were added. The mixture was allowed to react at room temperature for 2 hours to obtain a dark green solution. Subsequently, 0.175 g of Na_2_S_2_O_8_ (0.765 mmol) and 4 mL of water were added to the mixture, and the reaction was continued for another 2 hours. The reaction was then terminated by adding a large amount of water. A certain amount of dichloromethane was added to extract the mixture several times. A small amount of anhydrous magnesium sulfate was then added to the organic phase for drying. The filtered solution was dried to obtain the final product, DB·2BOB, which was used for subsequent experiments. Total ion chromatogram in the positive ESI mode of the DB·2BOB. m/z calcd for C_62_H_92_N_6_^+^ [M]^+^: 920.7378; found, 920.7433; calcd for C_62_H_92_N_6_^+^ [M]^2+^: 460.3686; found, 460.8746.

### Synthesis of DB·2BF_4_

In a 50 mL single-neck flask, 10 mL of hot DMF was added, followed by the addition of 0.94 g of N,N,N’,N’-tetrakis[4-(dibutylamino)phenyl]-p-phenylenediamine (1.02 mmol). The mixture was stirred at room temperature for 10 minutes to ensure complete dissolution. Next, 0.40 g of silver tetrafluoroborate (2.04 mmol), dissolved in 10 mL of DMF, was added to the flask, and the mixture was stirred at room temperature for an additional 30 minutes. The mixture was then filtered to obtain a dark green solution. Subsequently, the mixed solution was concentrated under vacuum to approximately 5 mL. To this concentrated solution, 50 mL of diethyl ether was added, and the mixture was allowed to precipitate in a refrigerator to form a solid. The solid was collected and washed with diethyl ether and then with petroleum ether. After drying, the final product, DB·2BF_4_, was obtained for subsequent experiments. Total ion chromatogram in the positive ESI mode of the DB·2BF_4_. m/z calcd for C_62_H_92_N_6_^+^ [M]^+^: 920.7378; found, 920.7433; calcd for C_62_H_92_N_6_^+^ [M]^2+^: 460.3686; found, 461.3773.

### Synthesis of DB·2X NPs

DB·2X (1 mg), dissolved in THF (2 mL), was slowly dripped into an aqueous solution of DSPE-PEG_2000_-COOH (5 mL, 1 mg mL^-1^). The mixture was then left open to the air and placed on a magnetic stirrer at room temperature, with the stirring speed adjusted to 700 rpm for overnight stirring. Subsequently, the tetrahydrofuran naturally evaporated. Finally, a DB·2TFSI NPs solution with a concentration of 200 μg mL^-1^ was obtained.

### Theoretical calculation

All DFT calculations were carried out by the Gaussian 16 program. Crystal structure optimization was calculated with the ONIOM method. For ONIOM calculations, the quantum mechanics (QM) region was calculated using the B3LYP method with the basis set of 6-31 G (d, p), and the molecular mechanics (MM) region was calculated using the Universal Force Field. The frequency was calculated at the same level as the geometry optimizations to guarantee the minimum without imaginary frequency. In addition, the electrostatic potential and the binding energy are also calculated at the same level in the cluster model. The reorganization energy was calculated using the Dushin package.

### Cell incubation

HUVEC cells, 4T1, and L929 cells originated from the American Type Culture Collection (ATCC). Cells were cultured in DMEM medium supplemented with 10% fetal bovine serum, 5% penicillin (100 mg/mL), and streptomycin (100 mg/mL), and maintained at 37 °C in an environment with 5% CO_2_. Cell density was determined using a cell counter. Cytotoxicity against HUVEC cells and L929 cells was assessed using the CCK-8 test. Briefly, HUVEC cells and L929 cells were seeded into 96-well plates at a density of 8×10^3^ cells per well and cultured in a humidified atmosphere at 37 °C with 5% CO_2_. These cells were then further incubated for 24 hours with fresh medium containing NPs at various concentrations. CCK-8 was added to each sample solution subsequently. The samples were then incubated for an additional hour in a humidified atmosphere at 37 °C with 5% CO_2_. Absorbance at a wavelength of 450 nm was measured using a microplate reader. Cell viability was calculated, and all data were expressed as mean ± SD compared to the untreated group.

### Animals

All experimental animal operations were approved by Zhejiang Provincial Hospital Animal Care and Use Committee (the approval number for in vivo experiments is 20240723ZDoa309w). The female BALB/c nude mice (~20 g, aged 8–10 weeks) and the female SD rats (~300 g, aged 12–14 weeks) involved in this work were provided from the Shanghai Slack Laboratory Animal Co., Ltd. All experimental animals were housed under standard conditions at the Experimental Animal Center of Zhejiang Provincial People’s Hospital and were bred and reared in specific pathogen-free conditions (a 12-h light/12-h dark cycle, room temperature at 25 °C, humidity at 50.0 ± 5.0%, with free access to food and water).

### In vitro PAI

To evaluate the performance of photoacoustic imaging, we employed various types and concentrations of DB·2X NPs to measure the intensity of PA signals and assess the linear relationship among these signals. Photoacoustic computed tomography (PACT) was conducted on the LOIS-3D system (TOMOWAVE, USA). Based on the needs, a tunable pulsed laser, capable of emitting light with wavelengths ranging from 660 to 2300 nanometers (Max output: 350 mJ per pulse, Pulse duration: 3–6 ns), is utilized as the light source, with 1064 nanometers, 1300 nanometers and 1700 nanometers being chosen for specific applications.

### In vivo PAI

The uterine cavity adhesion mouse model (*n* = 3) and the 4T1 (mouse breast cancer cells) subcutaneous tumor model (*n* = 3, the maximal tumor burden permitted by the institutional animal ethics committee was 800 mm^3^, and this limit was not exceeded in any animal during the study.) were anesthetized through inhalation of isoflurane. Subsequently, the mice were administered DB·2TFSI (1 mM, 200 μL) via vaginal route for the uterine cavity adhesion model and via tail vein injection for the subcutaneous tumor model. Scanning was then performed on the three-dimensional photoacoustic imaging system (LOIS-3D, TOMOWAVE LOIS-3D, TOMOWAVE Labs, USA) using pulsed excitation laser sources at 1064 nm and 1700 nm (Max output: 350 mJ per pulse, Pulse duration: 3–6 ns). Different PAI images were recorded. The imaging data were analyzed using Slicer imaging software (version 4.13). DB·2TFSI NPs were administered once at a concentration of 1 mM in a total volume of 200 μL per animal.

### Establish the mouse model of uterine cavity adhesions

The female mice were fasted for 12 hours before surgery but allowed free access to water. They received an intraperitoneal injection of 10% chloral hydrate (100 μL) under strict aseptic conditions. A longitudinal incision was made on the lower abdomen of the female mice to expose the uterus. Approximately 5 mm above the cervix, an incision of about 4 mm in length was made. A self-made uterine curette was used to perform curettage on the female mice, which was stopped when redness and swelling appeared on the four walls of the uterine cavity. The abdominal cavity was then rinsed with saline, and the abdominal wall of the female mice was sutured. A stable mouse model of intrauterine adhesions could be established 7 days later.

### In vivo biosafety assessment

To obtain a preliminary assessment of the in vivo biosafety of DB·2TFSI NPs, histopathological, hematological, and serum biochemical evaluations were performed in SD rats and BALB/c nude mice. Animals were randomly assigned to the control groups (*n* = 3) and DB·2TFSI NPs groups (*n* = 3). Animals in the treatment groups received a single tail-vein administration of DB·2TFSI NPs at a concentration of 1 mM, with a total injection volume of 400 μL per rat and 200 μL per mouse, whereas control animals received an equivalent volume of PBS. For histopathological evaluation, SD rats were euthanized at 3 days after administration, and the heart, liver, spleen, lung, and kidney were collected, fixed in 4% paraformaldehyde, embedded in paraffin, sectioned and stained with hematoxylin and eosin. For hematological and serum biochemical evaluation, blood samples were collected from BALB/c nude mice (*n* = 3) on days 7, 14, and 21 after administration. Whole-blood samples collected in EDTA-containing tubes were used for routine hematological analysis, whereas serum samples were used to assess hepatic and renal function.

### Reporting summary

Further information on research design is available in the Nature Portfolio Reporting Summary linked to this article.

## Supplementary information

Supplementary Information

Description of Additional Supplementary Files

Supplementary Movie 1-4

Reporting Summary

Transparent Peer Review file

## Source data

Souce Data

## Data Availability

All data in this study are included in the main text, supplementary information, and source data files. The single-crystal X-ray data related to DB·2TFSI, DB·2BOB, and DB·2BF_4_ have been deposited in the Cambridge Crystallographic Data Centre (CCDC) with deposition numbers 2403769, 2403770, and 2403783, respectively. These data can be obtained free of charge from the CCDC via www.ccdc.cam.ac.uk/data_request/cif. The current work places no restrictions on the availability of the data. Source data are provided with this paper.
