# Supplementary material for: Short Wavelength Infrared Diimmonium Nano-Agents for Photoacoustic Imaging
Source: Nat Commun. 2026 Oct 12;17:11562. doi: 10.1038/s41467-026-78613-7 (PMC13748559; doi:10.1038/s41467-026-78613-7)
Supplement: Supplementary file 1 [file 41467_2026_78613_MOESM1_ESM.pdf]

Short Wavelength Infrared Diimmonium Nano-Agents for Photoacoustic Imaging

Longcai Liu[1,2], Yang Chen[1], Liya Yu[1], Xiaozhou Mou[1]*, Guangxue Feng[2]*, Yu Cai[1]*

[1]Center for Rehabilitation Medicine, Rehabilitation & Sports Medicine Research Institute of Zhejiang Province, Department of Rehabilitation Medicine, Cancer Center, Zhejiang Provincial People's Hospital (Affiliated People's Hospital), Hangzhou Medical College, Hangzhou, Zhejiang, China, 310014.

[2]State Key Laboratory of Luminescent Materials and Devices, Guangdong Provincial Key Laboratory of Luminescence from Molecular Aggregates, School of Materials Science and Engineering, AIE Institute, South China University of Technology, Guangzhou, 510640, China.

*Corresponding authors: mouxz@zju.edu.cn, fenggx@scut.edu.cn, caiyu@hmc.edu.cn

Supplementary Figures

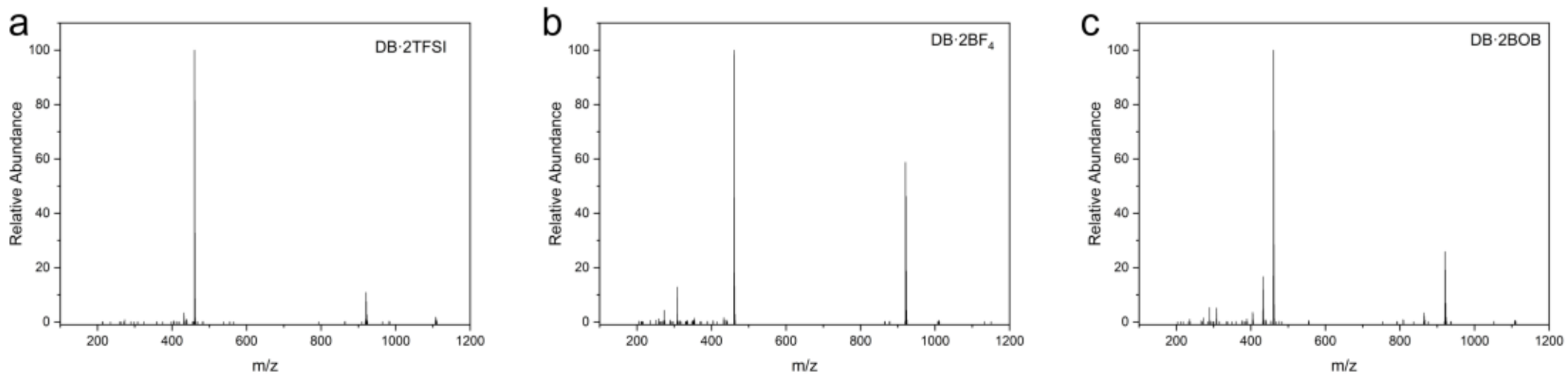

Supplementary Fig. 1. Mass spectrometric characterization of DB·2X. (a-c) Total ion chromatogram in the positive ESI mode of the DB·2X (X=$TFSI^-$, BF_4^-, BOB^-).

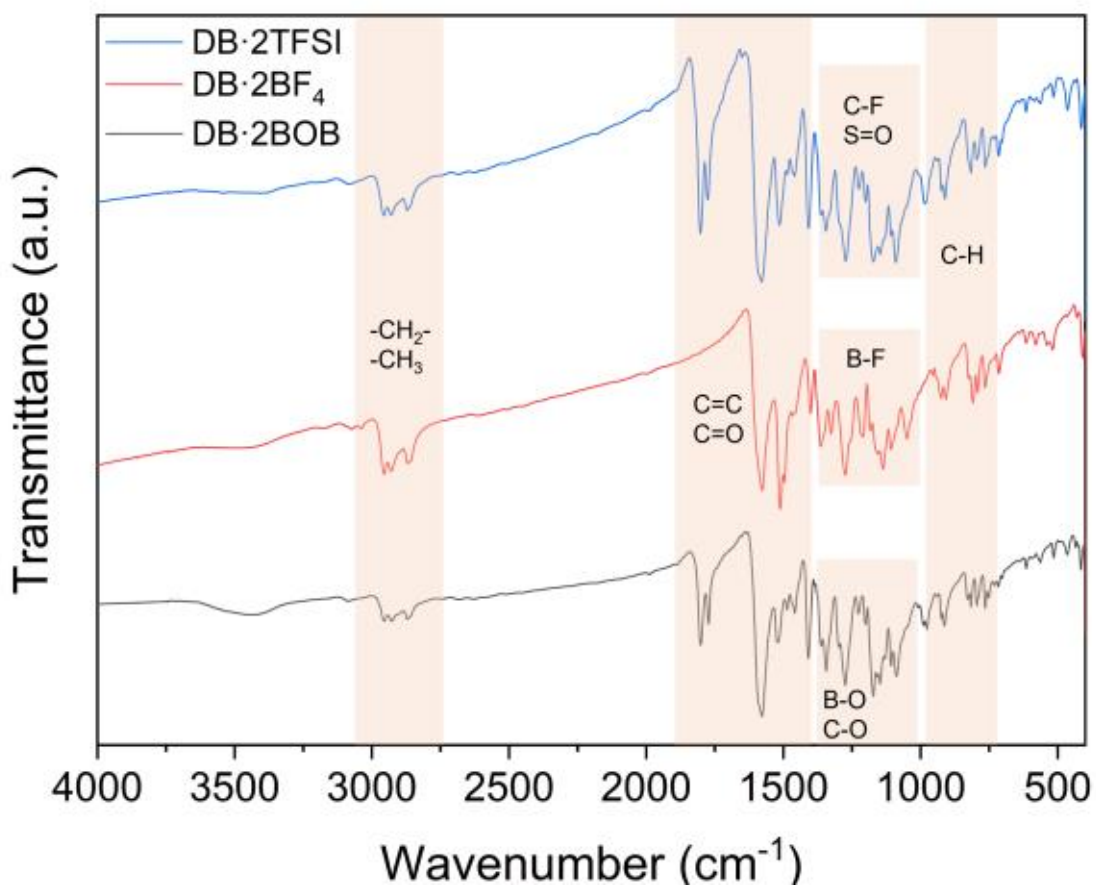

Supplementary Fig. 2. FT-IR characterization of DB·2X. FT-IR spectra of DB·2TFSI (blue), DB·$2BF_4$ (red) and DB·2BOB (black).

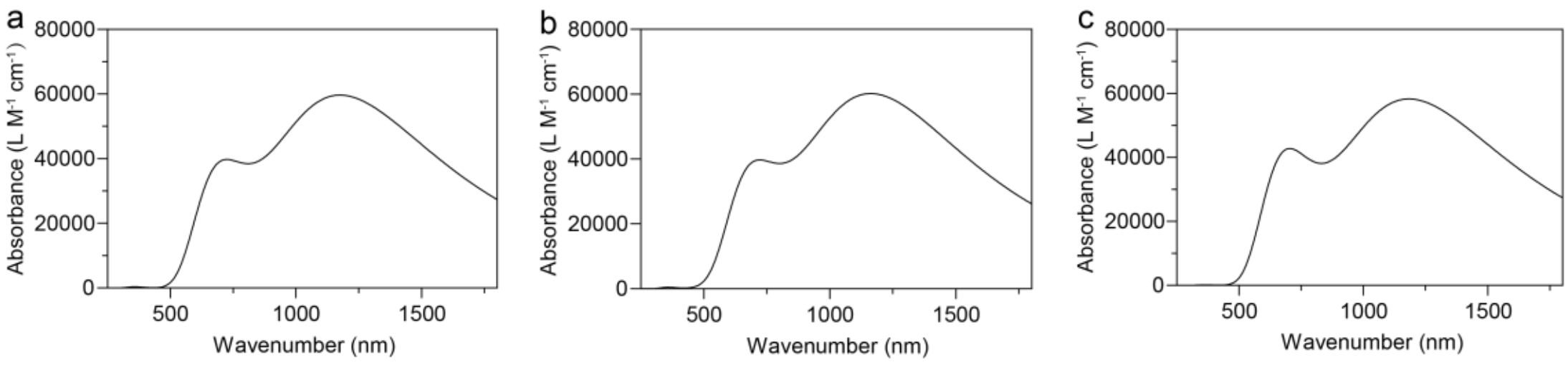

Supplementary Fig. 3. Theoretically calculated absorption spectra of DB·2X. (a-c) The theoretically calculated spectra of DB·2BF_4, DB·2TFSI, and DB·2BOB.

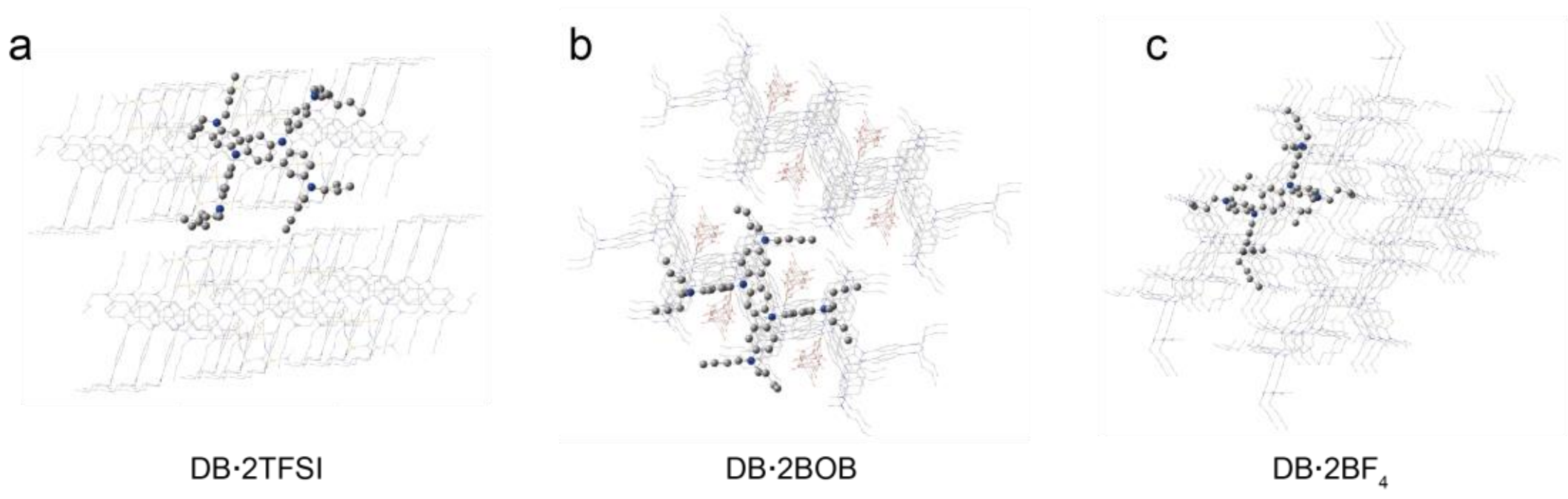

Supplementary Fig. 4. Optimized S_0-state conformations of DB·2X in the crystal phase. (a–c) Optimized S_0-state geometries of DB·2TFSI, DB·2BOB and DB·$2BF_4$ in the crystal phase.

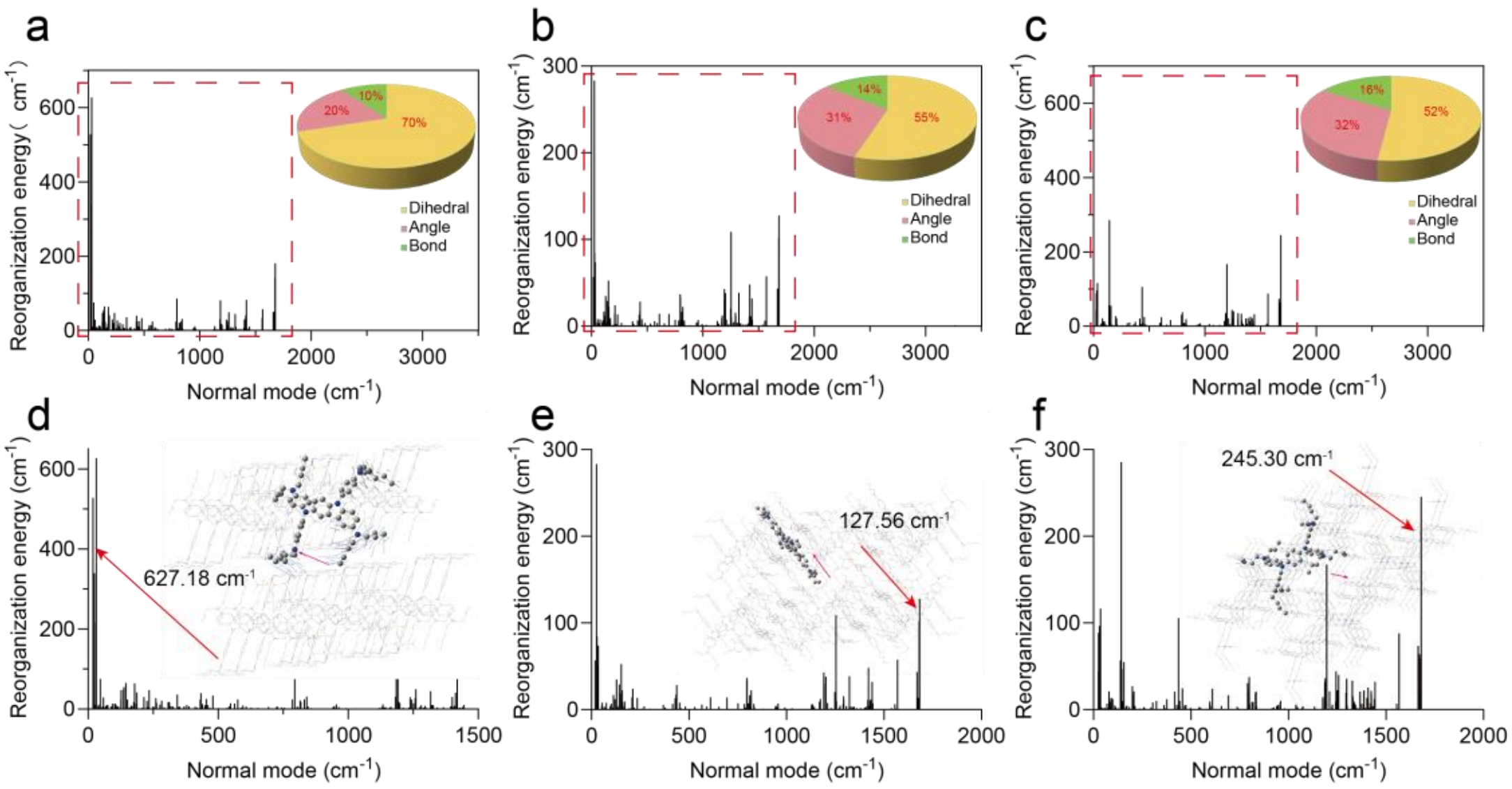

Supplementary Fig. 5. Vibrational contributions to the reorganization energy of DB·2X. Calculated total reorganization energy of (a-c). DB·2TFSI, DB·2BOB and DB·2BF$_4$ at normal mode frequency and contribution of different vibration modes to the total reorganization energy. In the crystalline phase, the reorganization energy of (d-f). DB·2TFSI, DB·2BOB and DB·2BF$_4$. with representative vibrational modes varies with the frequency of the forward mode.

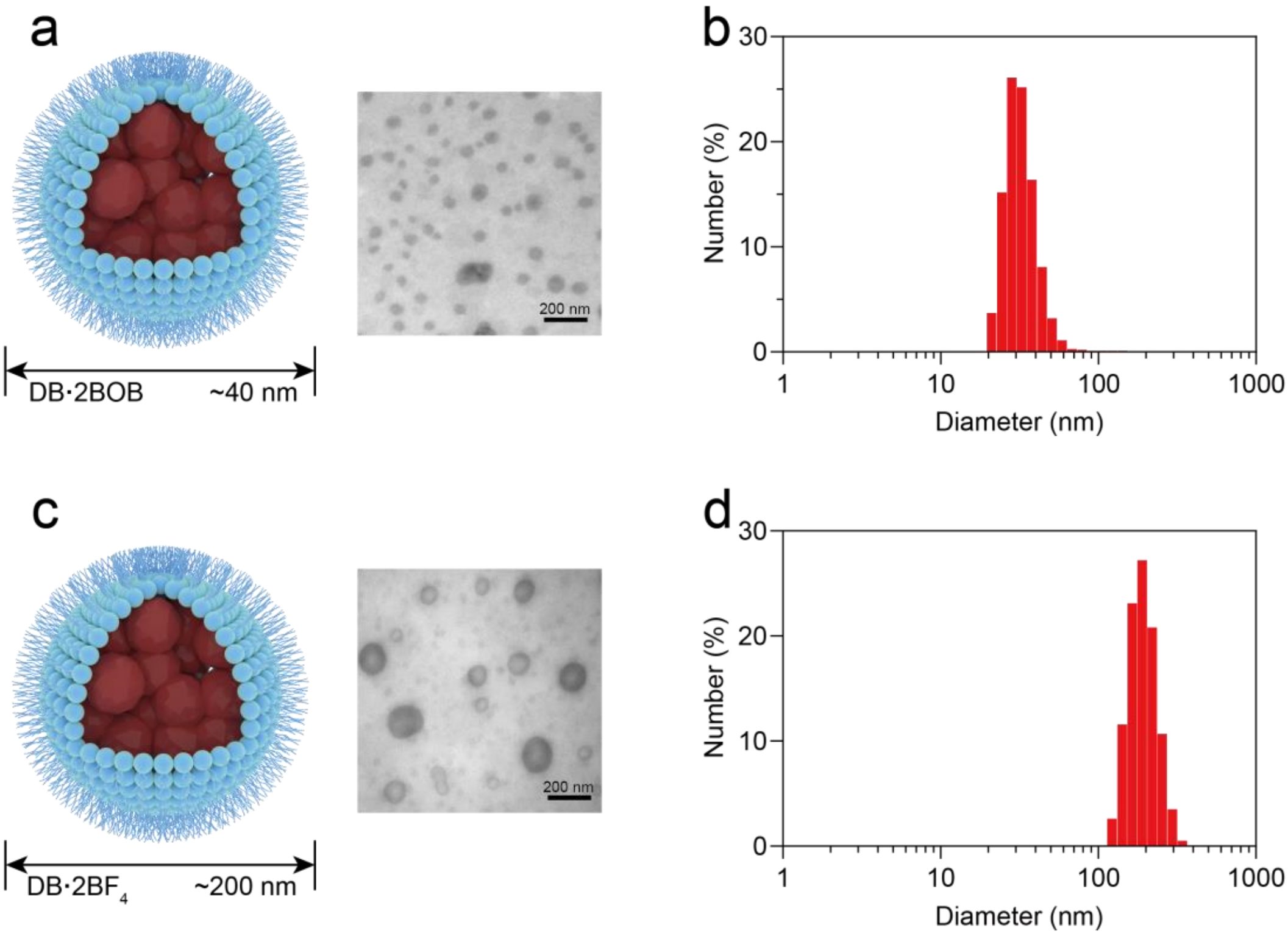

Supplementary Fig. 6. Morphological and size characterization of DB·2BOB and DB·2BF_4 nanoparticles. (a) Representative TEM image of DB·2BOB. (b) DLS size distribution of DB·2BOB. (c) Representative TEM image of DB·2BF_4. (d) DLS size distribution of DB·2BF_4.

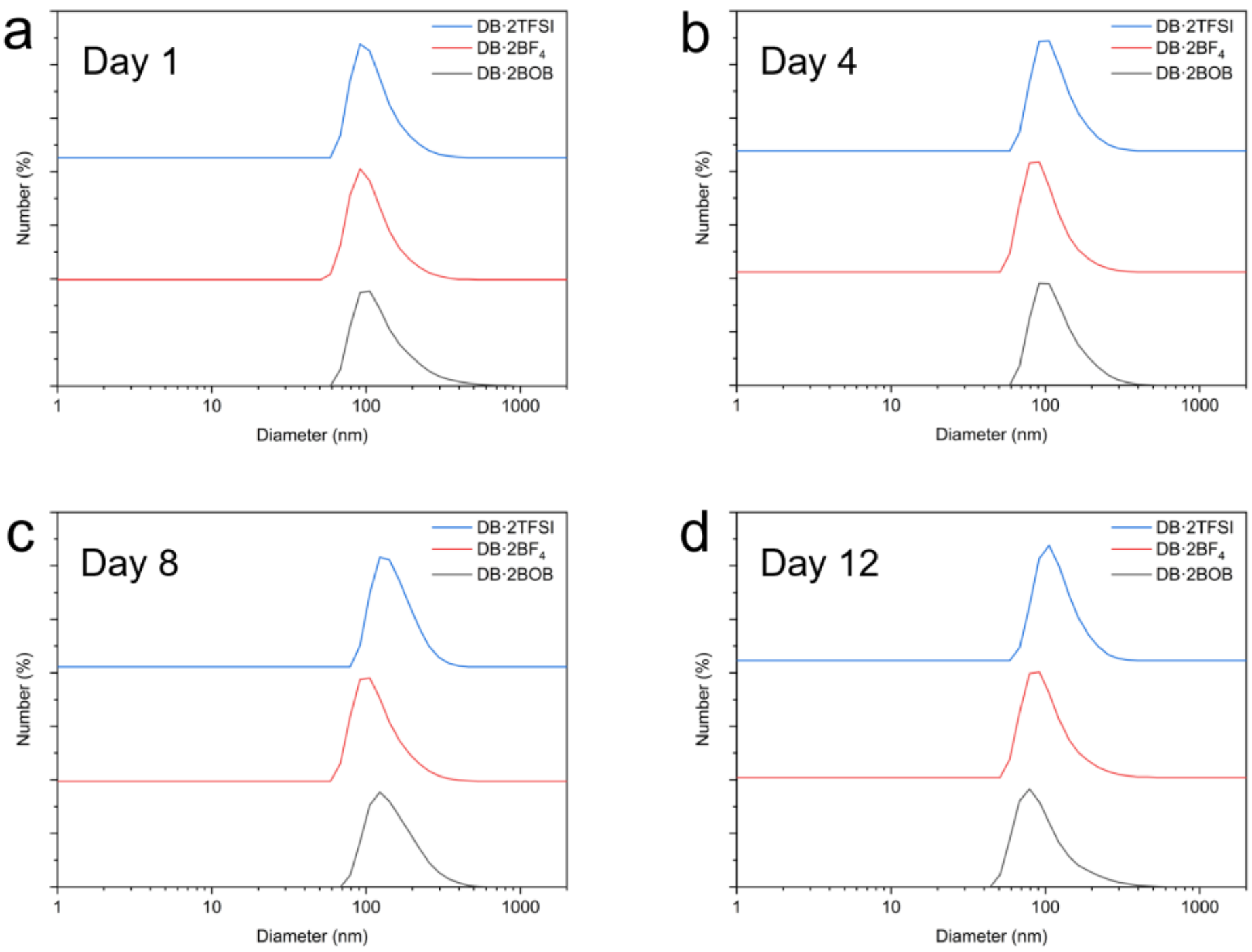

Supplementary Fig. 7. Long-term size stability of DB·2X nanoparticles. (a-d) Particle size of DB·2X NPs (X=TFSI$^-$, BF_4^-, BOB$^-$) on different days.

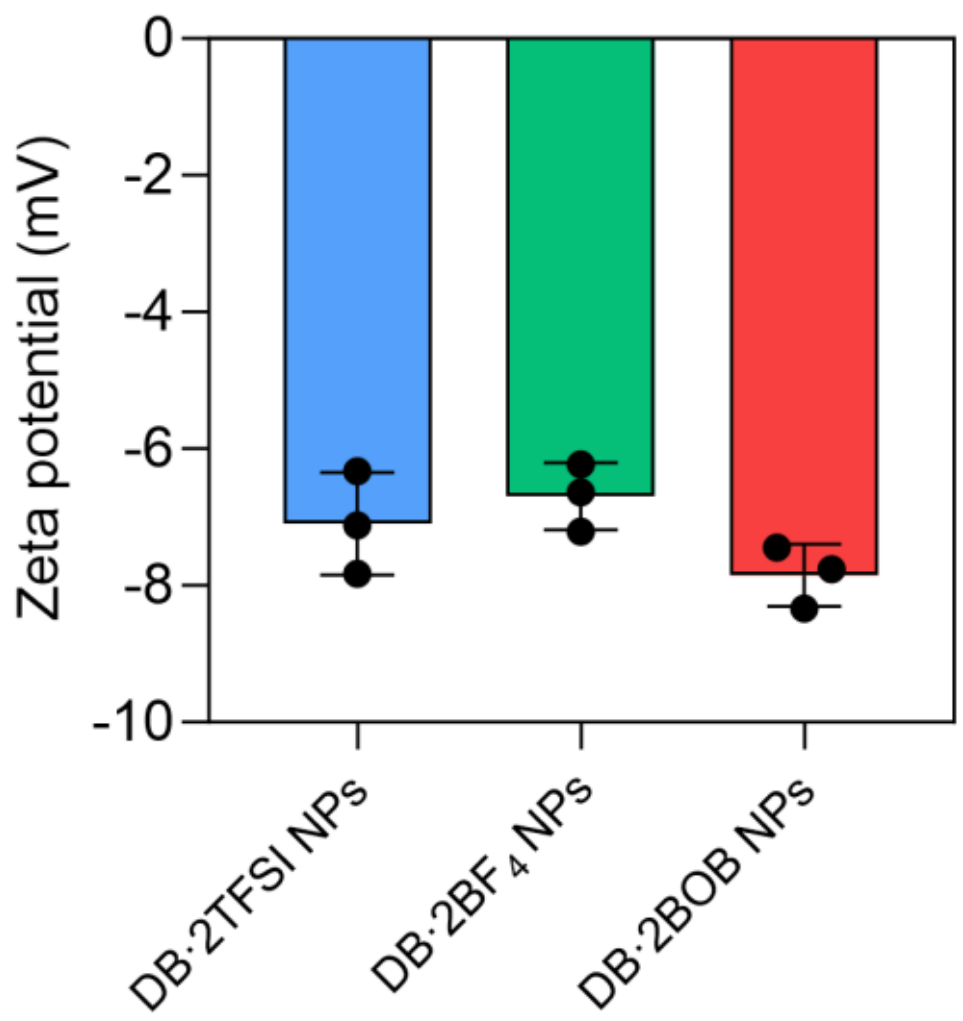

Supplementary Fig. 8. Zeta potentials of DB·2X nanoparticles. Zeta potential size of DB·2X (X=TFSI$^-$, BF_4^-, BOB$^-$). Data are presented as mean ± SD from n = 3 independent experiments. Individual dots represent independent experiments.

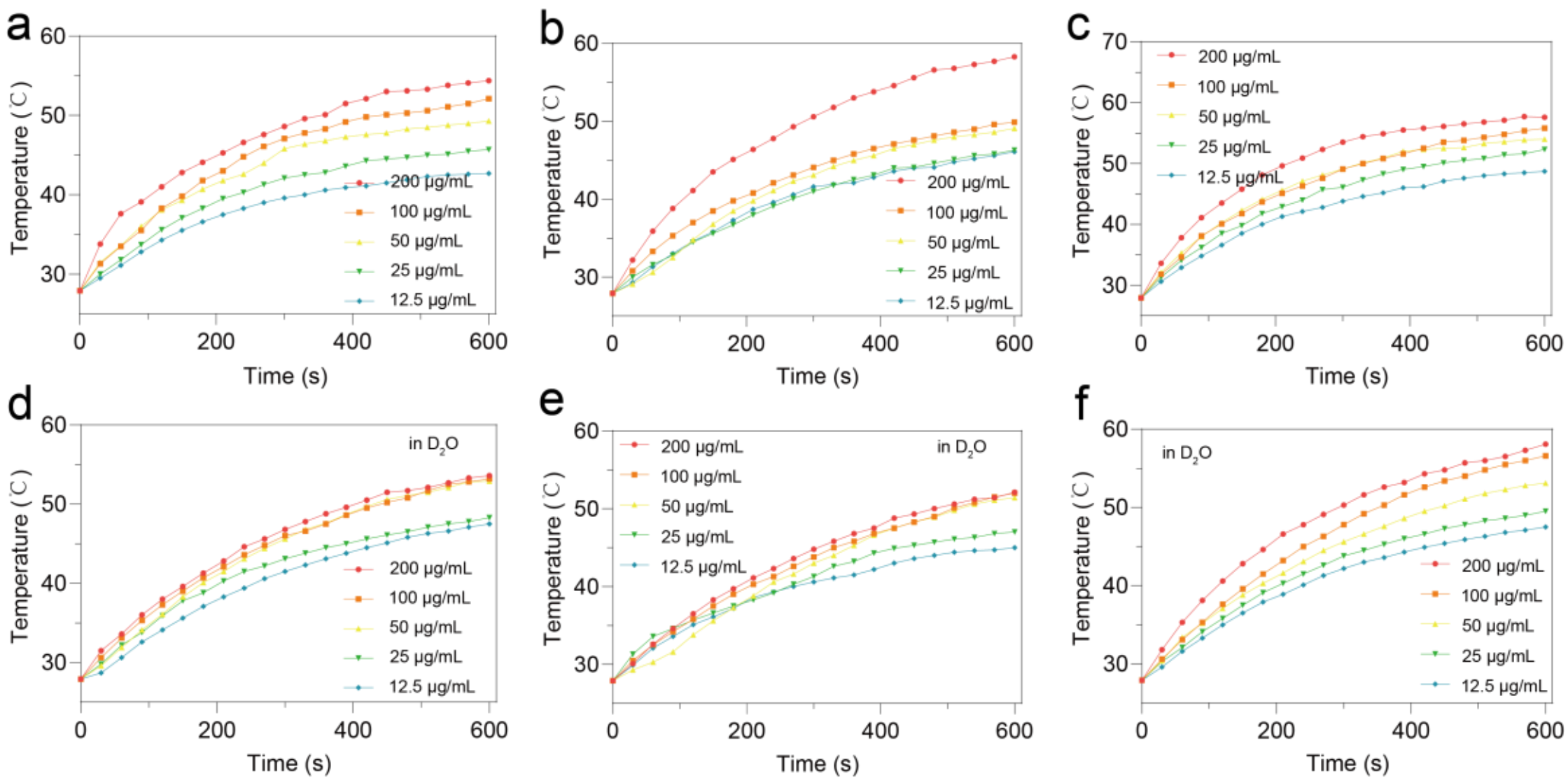

Supplementary Fig. 9. Concentration-dependent photothermal performance of DB·2X nanoparticles. Temperature increases of (a) DB·2TFSI NPs, (b) DB·2BOB and (c) DB·$2BF_4$ at different concentrations under 1064 nm laser irradiation (power of 1.0 W/cm^2). Temperature increase of (d) DB·2TFSI NPs, (e) DB·2BOB and (f) DB·$2BF_4$. at different concentrations under 1550 nm laser irradiation (power of 1.0 W/cm^2).

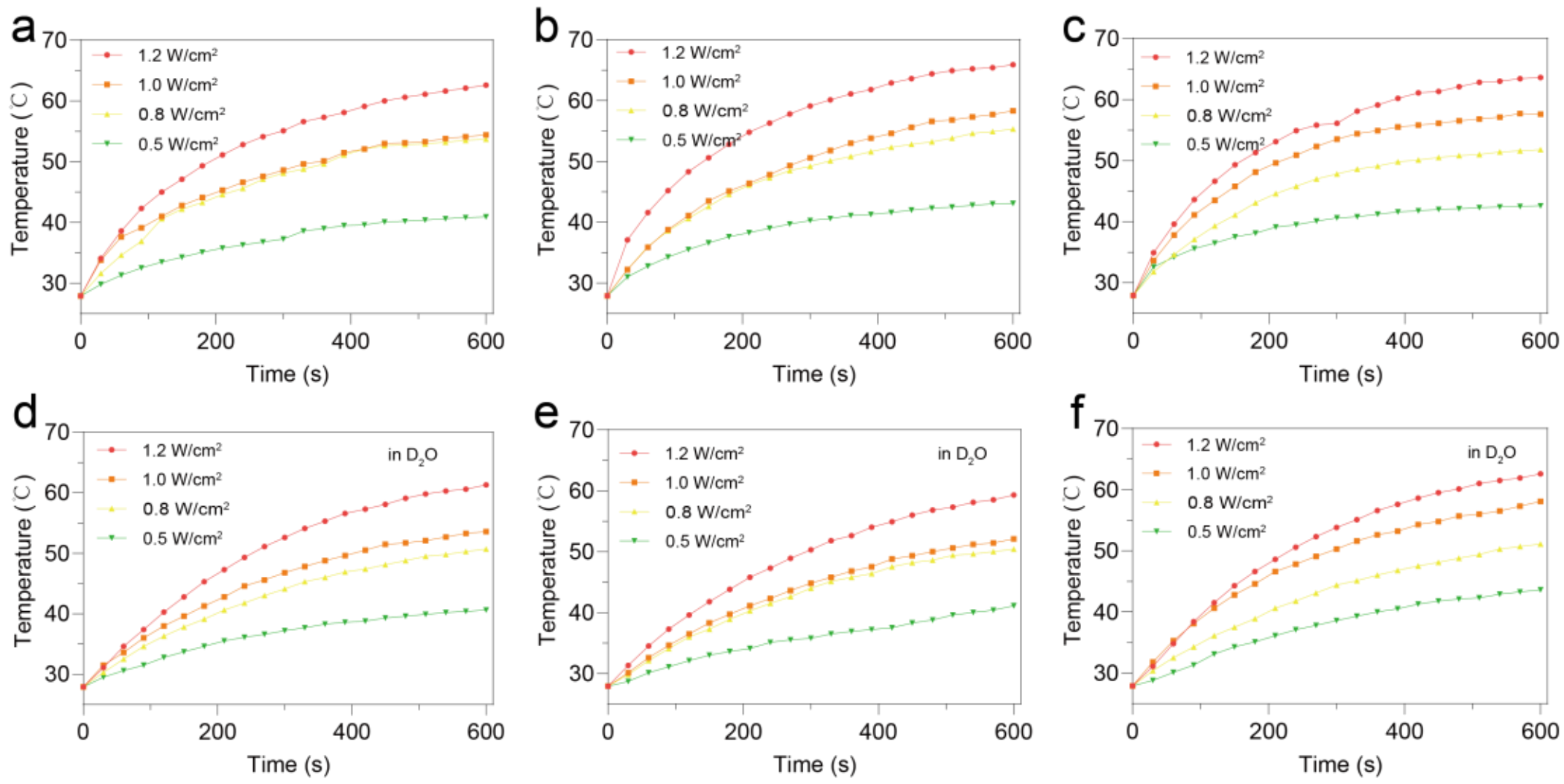

Supplementary Fig. 10. Laser-power-dependent photothermal performance of DB·2X nanoparticles. Temperature increases of (a) DB·2TFSI NPs, (b) DB·2BOB and (c) DB·$2BF_4$ at different laser power under 1064 nm laser irradiation. Temperature increase of (d). DB·2TFSI NPs, (e) DB·2BOB and (f) DB·$2BF_4$. at different laser power under 1550 nm laser irradiation.

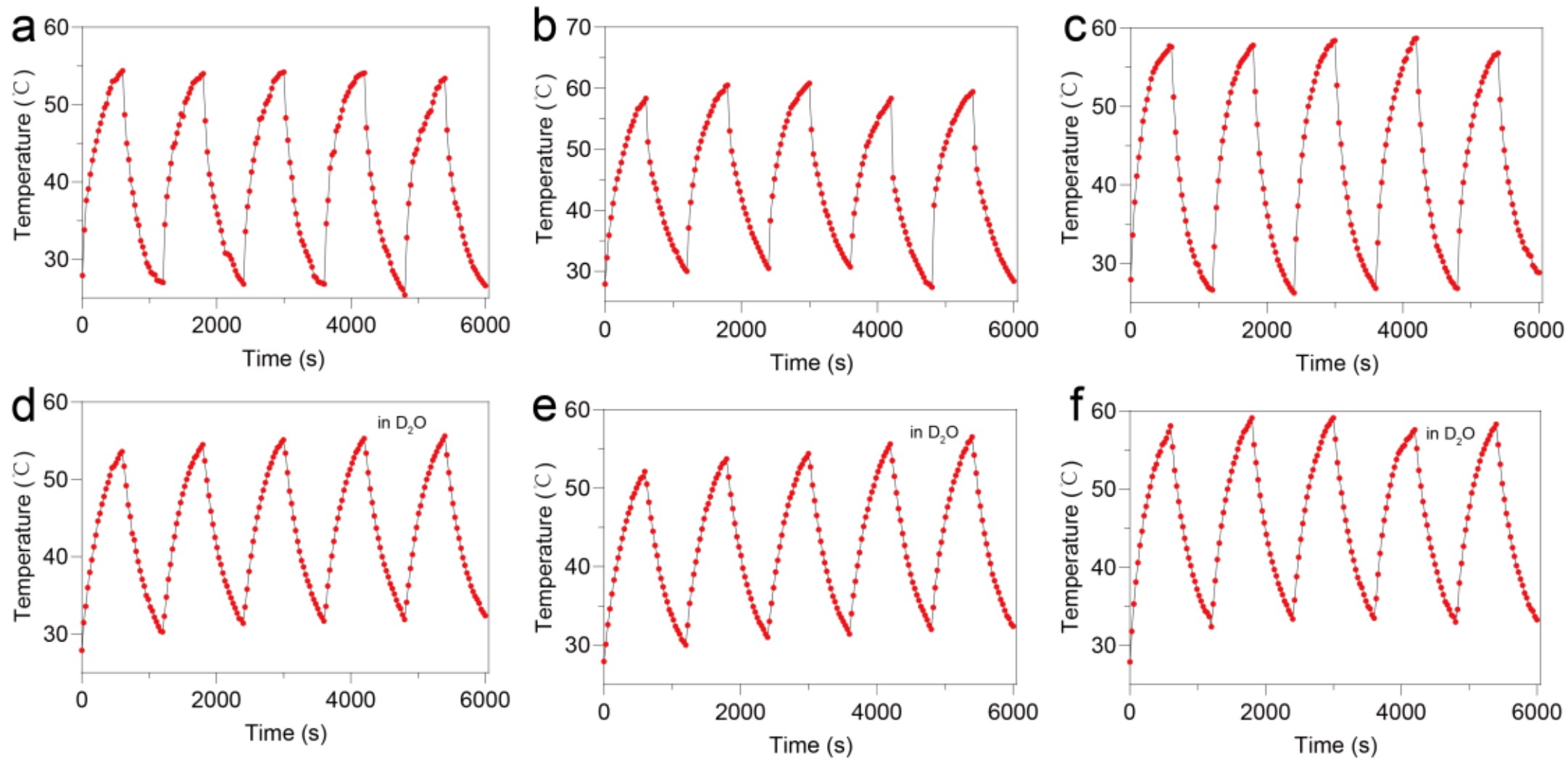

Supplementary Fig. 11. Photothermal cycling stability of DB·2X nanoparticles. Temperature rise and fall cycles curve of (a) DB·2TFSI NPs, (b) DB·2BOB and (c) DB·$2BF_4$ over five cycles, under 1064 nm laser irradiation at a power of 1.0 W/cm^2. Temperature rise and fall cycles curve of (d) DB·2TFSI NPs, (e) DB·2BOB and (f) DB·$2BF_4$ over five cycles, under 1550 nm laser irradiation at a power of 1.0 W/cm^2.

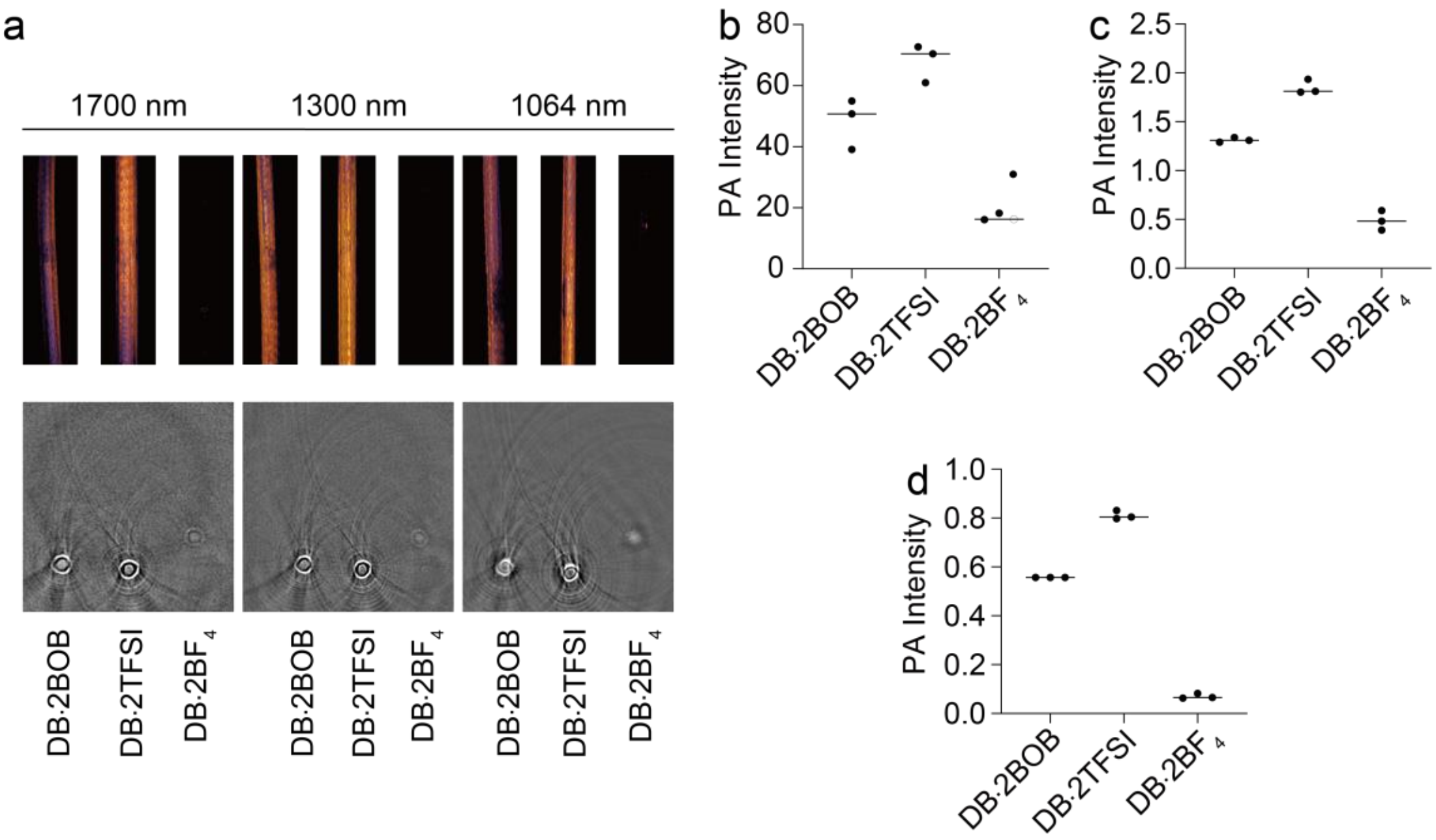

Supplementary Fig. 12. Wavelength-dependent in vitro photoacoustic performance of DB·2X nanoparticles. (a) PA signal of different NPs different pulsed laser excitation wavelengths (1064 nm, 1300 nm, and 1700 nm). (b-d) PA intensity derived from (a) (Excitation laser source: b: 1064 nm, c: 1300 nm, and d: 1700 nm).

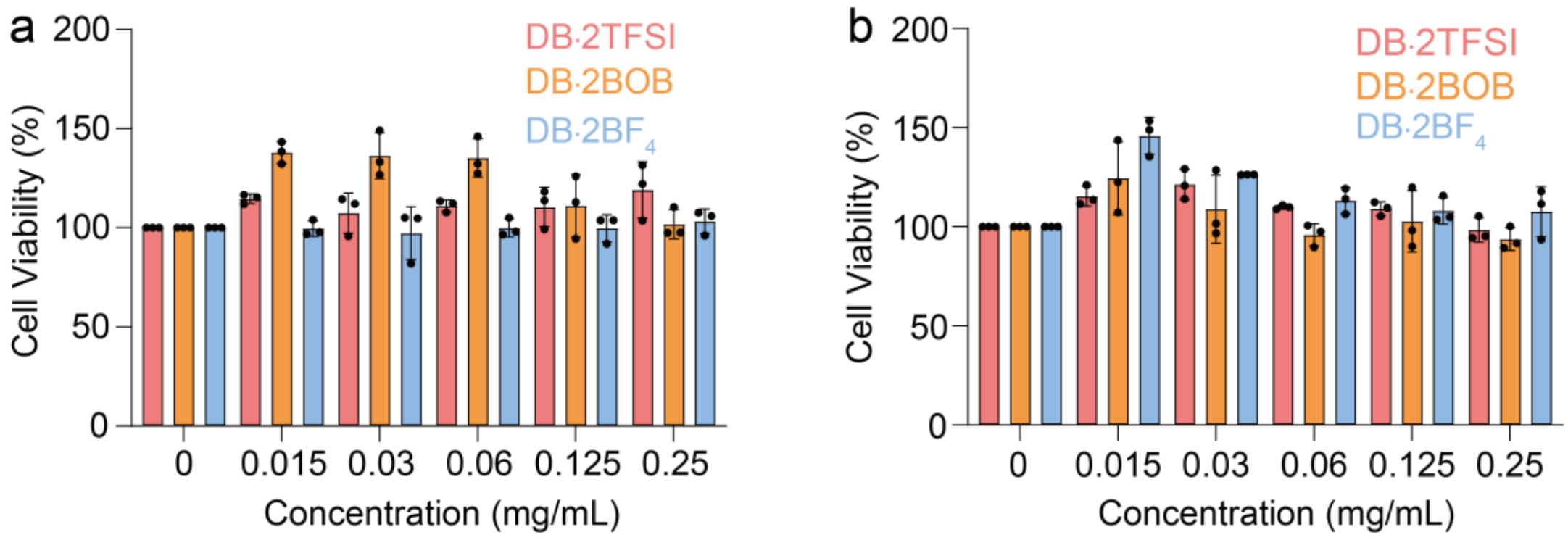

Supplementary Fig. 13. Cytocompatibility of DB·2X nanoparticles in HUVEC and L929 cells. (a) Toxicity of different NPs towards HUVEC cells. (b) Toxicity of different NPs towards L929 cells. Data are presented as mean ± SD from n = 3 independent experiments. Individual dots represent independent experiments.

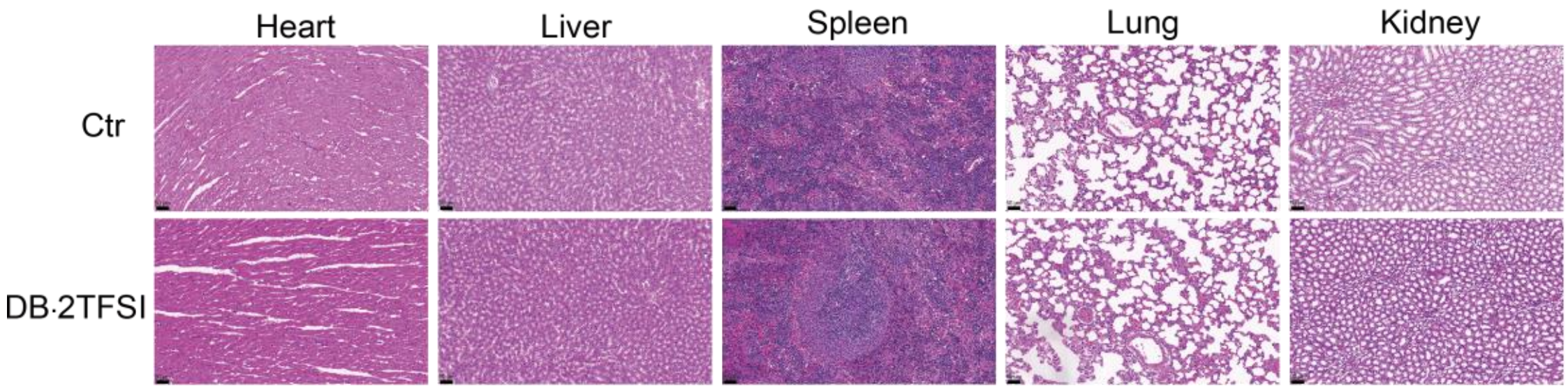

Supplementary Fig. 14. Histopathological biosafety evaluation of DB·2TFSI nanoparticles. H&E stained images of major organs slices (Group: Ctr and DB·2TFSI). Scale bar: 50 μm.

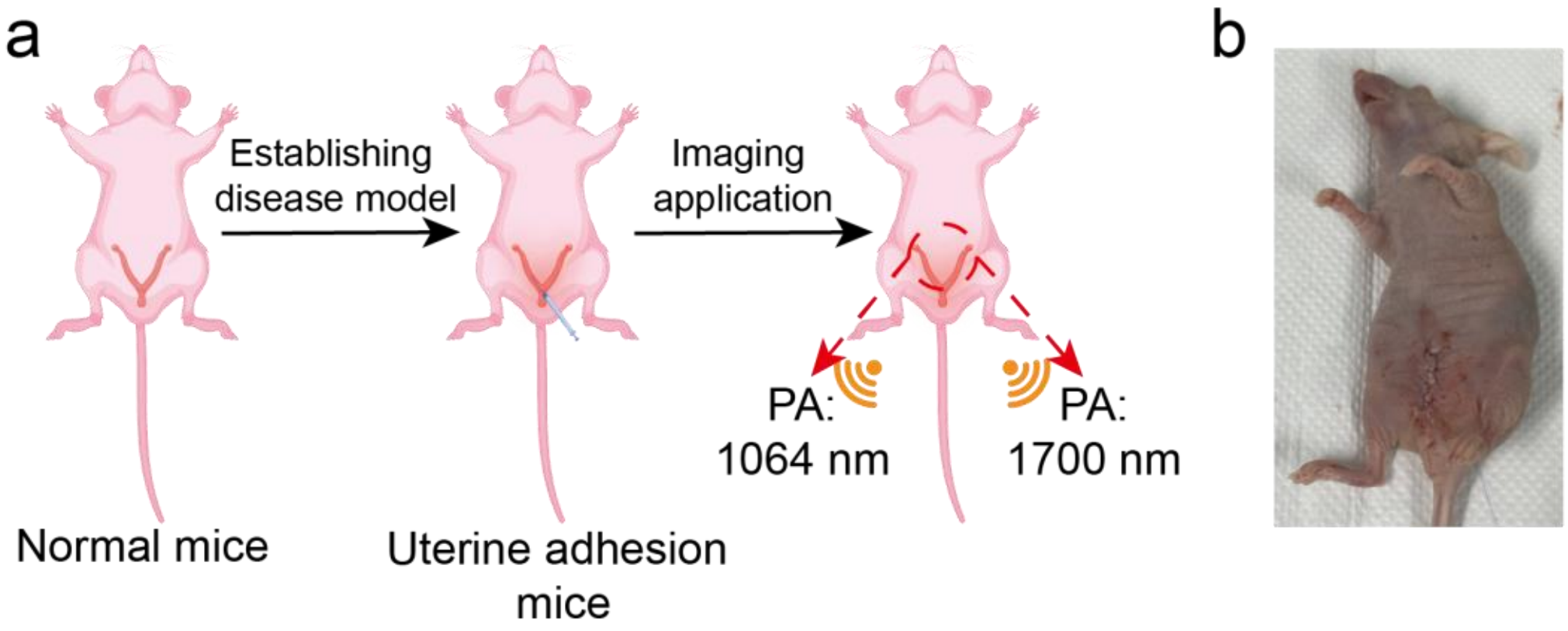

Supplementary Fig. 15. Establishment of the mouse model of uterine cavity adhesions. PAI of uterine adhesions. (a) Schematic illustration of the surgical procedure used to establish uterine cavity adhesions. Created in BioRender. Mou, X. (2026) https://BioRender.com/tfs6638 (b) Representative image of a mouse after abdominal suturing following surgery.

Supplementary Figures

Supplementary Table 1. Total interaction energy between different ligand and the parent.

Counter anion	Binding Energy (kcal/moL)
**BF_4^-**	87.8
**$TFSI^-$**	108.0
**BOB^-**	104.4

Supplementary Table 2. Crystal data and structure refinement for DB·2BOB, DB·2TFSI and DB·$2BF_4$.

	**DB·2BOB**	**DB·2TFSI**	**DB·2BF$_4$**
**CCDC**	2403770	2403769	2403783
**Empirical formula**	$C_{72}H_{100}B_2Cl_4N_6O_{18}$	$C_{66}H_{92}F_{12}N_8O_8S_4$	$C_{62}H_{92}BF_4N_6$
**Formula weight**	1500.99	1481.71	1008.22
**Temperature/K**	193.00	233.00	303.00
**Crystal system**	Monoclinic	Triclinic	Triclinic
**Space group**	$P2_1/c$	P-1	P-1
**a/Å**	18.105 (2)	10.9150 (17)	14.937 (6)
**b/Å**	10.6520 (11)	10.987 (3)	15.113 (6)
**c/Å**	21.780 (3)	16.520 (3)	15.424 (6)
**α/°**	90	79.171 (8)	74.345 (10)
**β/°**	109.214 (5)	87.708 (6)	69.631 (11)
**γ/°**	90	86.896 (7)	74.802 (11)
**Volume/Å^3**	3966.5 (8)	1942.1 (7)	3088 (2)
**Z**	2	1	2
**ρ_{calc}g/cm^3**	1.257	1.267	1.084
**μ/mm^{-1}**	1.246	0.205	0.071
**F (000)**	1592.0	780.0	1094.0
**Crystal size/mm^3**	0.13×0.1×0.08	0.12×0.11×0.08	0.12×0.1×0.09
**Radiation**	Ga Kα (λ=1.34139)	MoKα (λ=0.71073)	MoKα (λ=0.71073)
**2θ range for data collection**	7.35 to 107.804	4.44 to 52.914	4.428 to 50.052
**Index range**	$-21\le h\le 19$, $-9\le k\le 12$, $-24\le l\le 26$	$-12\le h\le 13$, $-13\le k\le 13$, $-20\le l\le 20$	$-16\le h\le 17$, $-17\le k\le 17$, $0\le l\le 18$
**Reflections collected**	36102	40491	9867
**Independent reflections**	7233 [R_{int}=0.1105,	7932 [R_{int}=0.0639,	9867 [R_{int}=?,

	R_{sigma}=0.0815]	R_{sigma}=0.0433]	R_{sigma}=0.1639]
**Data/restraints/parameters**	7233/423/456	7932/86/486	9867/703/742
**Goodness-of-fit on F^2**	1.010	1.056	0.976
**Final R indexes [I>=2σ(I)]**	R_1=0.1251, wR_2=0.3143	R_1=0.0733, wR_2=0.2137	R_1=0.1337, wR_2=0.2768
**Final R indexes [all data]**	R_1=0.1835, wR_2=0.3484	R_1=0.1103, wR_2=0.2499	R_1=0.2669, wR_2=0.3490
**Largest diff. peak/hole/e Å^{-3}**	0.48/-0.40	0.48/-0.37	0.33/-0.32

Supplementary Table 3. Blood routine test of mice at different days after injection of DB·2TFSI NPs (n=3).

**Test items**	**DAY 7 (n=3)**			**DAY 14 (n=3)**			**DAY 21 (n=3)**		
WBC	5.13	4.87	4.88	4.92	4.85	8	4.95	4.68	4.39
RBC	7.94	9.18	9.7	10.58	9.93	10.15	10.3	10.74	8.79
HGB	147	152	164	194	162	175	199	203	152
MCV	47.8	51	52.1	53.4	49.5	51.5	54.7	55.1	52.4
PLT	498	526	555	533	560	526	433	601	457
Lym#	3.39	3.29	3.21	3.43	3.5	4.62	3.38	1.97	2.96
Mid#	0.33	0.28	0.31	0.35	0.33	0.51	0.31	0.39	0.25
GR#	1.41	1.3	1.36	1.14	1.02	2.87	1.26	2.32	1.18
Lym%	66.1	67.5	65.8	69.7	72.1	57.8	68.2	42.2	67.4
Mid%	6.4	5.8	6.3	7.2	6.8	6.4	6.3	8.3	5.7
GR%	27.5	26.7	27.9	23.1	21.1	35.8	25.5	49.5	26.9
P_LCR	56.4	56.8	34.6	54	28.1	32.7	62.7	13.8	37
P_LCC	281	299	192	288	157	172	272	83	169
HCT	38	46.8	50.5	56.5	49.2	52.2	56.3	59.2	46.1
MCHC	387	325	325	343	329	335	353	343	330
RDW_CV	12.8	10.9	11.8	12.8	11.2	11.7	13.4	13.6	11.6
RDW_SD	66.6	60.8	61	60.8	57.1	63.3	62.7	66.1	57.4
MCH	18.5	16.6	16.9	18.3	16.3	17.2	19.3	18.9	17.3
MPV	9	9.2	9.5	9.6	9.1	9.3	9.7	9.7	9.4
PCT	0.448	0.484	0.527	0.512	0.51	0.489	0.42	0.583	0.43
PDW	17.1	17.1	17.6	16.8	17.1	16.8	16.8	17.7	18

Supplementary Table 4. Liver function indicators and renal function indicators of mice at different days after injection of DB·2TFSI NPs (n=3).

**Test items**	**DAY 7**			**DAY 14**			**DAY 21**		
ALT (U/L)	34.7	31.9	28.0	39.6	38.7	31.8	35.5	37.5	38.8
AST (U/L)	105.4	101.2	108.5	186.5	174.1	100.0	210.3	206.3	165.6
CREA (umol/L)	12.2	14.6	16.0	18.4	18.1	13.4	15.9	15.8	16.0
BUN (mmol/L)	2.519	2.747	2.752	2.346	2.318	2.519	3.866	3.265	2.500
