# Supplementary material for: Short Wavelength Infrared Diimmonium Nano-Agents for Photoacoustic Imaging
Source: Nat Commun. 2026 Oct 12;17:11562. doi: 10.1038/s41467-026-78613-7 (PMC13748559; doi:10.1038/s41467-026-78613-7)
Supplement: Supplementary file 2 [file 41467_2026_78613_MOESM2_ESM.pdf]

Description of Additional Supplementary Files

Supplementary Movie 1. 1064 nm PAI of uteri.

Supplementary Movie 2. 1700 nm PAI of uteri.

Supplementary Movie 3. 1064 nm PAI of tumor.

Supplementary Movie 4. 1700 nm PAI of tumor.
