# Supplementary material for: Short Wavelength Infrared Diimmonium Nano-Agents for Photoacoustic Imaging
Source: Nat Commun. 2026 Oct 12;17:11562. doi: 10.1038/s41467-026-78613-7 (PMC13748559; doi:10.1038/s41467-026-78613-7)
Supplement: Supplementary file 3 [file 41467_2026_78613_MOESM4_ESM.pdf]

nature portfolio

Corresponding author(s): Yu Cai; Guangxue Feng; Xiaozhou Mou

Last updated by author(s): Mar 20, 2026

Reporting Summary

Nature Portfolio wishes to improve the reproducibility of the work that we publish. This form provides structure for consistency and transparency in reporting. For further information on Nature Portfolio policies, see our Editorial Policies and the Editorial Policy Checklist.

Statistics

For all statistical analyses, confirm that the following items are present in the figure legend, table legend, main text, or Methods section.

n/a	Confirmed	
☐	☒	The exact sample size (n) for each experimental group/condition, given as a discrete number and unit of measurement
☐	☒	A statement on whether measurements were taken from distinct samples or whether the same sample was measured repeatedly
☒	☐	The statistical test(s) used AND whether they are one- or two-sided<br>*Only common tests should be described solely by name; describe more complex techniques in the Methods section.*
☒	☐	A description of all covariates tested
☒	☐	A description of any assumptions or corrections, such as tests of normality and adjustment for multiple comparisons
☒	☐	A full description of the statistical parameters including central tendency (e.g. means) or other basic estimates (e.g. regression coefficient) AND variation (e.g. standard deviation) or associated estimates of uncertainty (e.g. confidence intervals)
☒	☐	For null hypothesis testing, the test statistic (e.g. F, t, r) with confidence intervals, effect sizes, degrees of freedom and P value noted<br>*Give P values as exact values whenever suitable.*
☒	☐	For Bayesian analysis, information on the choice of priors and Markov chain Monte Carlo settings
☒	☐	For hierarchical and complex designs, identification of the appropriate level for tests and full reporting of outcomes
☒	☐	Estimates of effect sizes (e.g. Cohen's d, Pearson's r), indicating how they were calculated

Our web collection on statistics for biologists contains articles on many of the points above.

Software and code

Policy information about availability of computer code

Data collection	The UV-NIR absorption spectra of the compounds were recorded using a UV-3600 Plus spectrophotometer. The relative molecular weight of the compound was determined by Thermo Fisher Scientific Orbitrap Exploris 120. The FT-IR spectra of the compound were detected using a Thermo Fisher Scientific Nicolet iS20 instrument. The particle size distribution of the NPs was evaluated using a Zetasizer nano ZS particle analyzer (Malvern Instruments Ltd.). TEM images were recorded with a JEM 2100F microscope. Single-crystal X-ray diffraction (SXRD) analysis was conducted using a Bruker smart Apex with a molybdenum target at room temperature, collecting single crystals of different compounds at a scan rate of 5° min^{-1}. The crystal structures were solved using Olex2 software. In vitro and in vivo PAI were collected by TOMOWAVE LOIS-3D (USA).
Data analysis	The imaging data were analyzed using Slicer imaging software (version 4.13). Quantitative analysis of photoacoustic images was performed using Image J software (version 1.6.0, National Institutes of Health, USA). The data was analyzed using OriginPro2020 (64 bit), GraphPad Prism 10 (USA), and Olex2. Density functional theory calculations of molecular forms have been completed through the Gaussian16 project. The reorganization can be calculated through the Dushin software package.

For manuscripts utilizing custom algorithms or software that are central to the research but not yet described in published literature, software must be made available to editors and reviewers. We strongly encourage code deposition in a community repository (e.g. GitHub). See the Nature Portfolio guidelines for submitting code & software for further information.

Data

Policy information about availability of data

All manuscripts must include a data availability statement. This statement should provide the following information, where applicable:

- Accession codes, unique identifiers, or web links for publicly available datasets
- A description of any restrictions on data availability
- For clinical datasets or third party data, please ensure that the statement adheres to our policy

The authors declare that all data from this study are available in the supplementary information and source data. The data supporting the findings of this study are available within the paper and its supplementary information, and from the corresponding author upon reasonable request.

Research involving human participants, their data, or biological material

Policy information about studies with human participants or human data. See also policy information about sex, gender (identity/presentation), and sexual orientation and race, ethnicity and racism.

Reporting on sex and gender	Not applicable
Reporting on race, ethnicity, or other socially relevant groupings	Not applicable
Population characteristics	Not applicable
Recruitment	Not applicable
Ethics oversight	Not applicable

Note that full information on the approval of the study protocol must also be provided in the manuscript.

Field-specific reporting

Please select the one below that is the best fit for your research. If you are not sure, read the appropriate sections before making your selection.

☒ Life sciences ☐ Behavioural & social sciences ☐ Ecological, evolutionary & environmental sciences

For a reference copy of the document with all sections, see nature.com/documents/nr-reporting-summary-flat.pdf

Life sciences study design

All studies must disclose on these points even when the disclosure is negative.

Sample size	According to the principle of replication, for nanoparticles, cell testing, and animal experiments, the sample size (n) is controlled to be at least three. The number of biological replicates required to ensure statistical significance. For details, please refer to the figure legends.
Data exclusions	No data were excluded from the data analysis.
Replication	Experiments were repeated in at least three independent experiments with similar results. All experiments were reproduced to reliably support the conclusions stated in the manuscript.
Randomization	All samples were randomly allocated.
Blinding	Researchers knew nothing about group allocation during the data collection and analysis process.

Reporting for specific materials, systems and methods

We require information from authors about some types of materials, experimental systems and methods used in many studies. Here, indicate whether each material, system or method listed is relevant to your study. If you are not sure if a list item applies to your research, read the appropriate section before selecting a response.

Materials & experimental systems

n/a	Involved in the study
☒	☐ Antibodies
☐	☒ Eukaryotic cell lines
☒	☐ Palaeontology and archaeology
☐	☒ Animals and other organisms
☒	☐ Clinical data
☒	☐ Dual use research of concern
☒	☐ Plants

Methods

n/a	Involved in the study
☒	☐ ChIP-seq
☒	☐ Flow cytometry
☒	☐ MRI-based neuroimaging

Eukaryotic cell lines

Policy information about cell lines and Sex and Gender in Research

Cell line source(s)	4T1, HUVEC cell and L929 cells were purchased from American Type Culture Collection (ATCC) and maintained in the laboratory.
Authentication	These cell lines were authenticated by the supplier using STR analysis.
Mycoplasma contamination	No contamination was detected.
Commonly misidentified lines (See ICLAC register)	These cell lines that we used were not listed in commonly misidentified lines in ICLAC Register.

Animals and other research organisms

Policy information about studies involving animals; ARRIVE guidelines recommended for reporting animal research, and Sex and Gender in Research

Laboratory animals	Balb/c nude mice and SD rats were purchased from Shanghai Slack Laboratory Animal Co., Ltd.
Wild animals	No wild animals were used in this study.
Reporting on sex	There was no other sex bias in the animals used.
Field-collected samples	The study did not involve samples collected from the field.
Ethics oversight	All animal experiments were conducted under the guidance and regulations of the Chinese Law on Institutional Animal Care and Use. The entire experiment was approved by the Animal Welfare Ethics Committee of Zhejiang Provincial People's Hospital.

Note that full information on the approval of the study protocol must also be provided in the manuscript.

Plants

Seed stocks	Not applicable
Novel plant genotypes	Not applicable
Authentication	Not applicable
