# Supplementary material for: Short Wavelength Infrared Diimmonium Nano-Agents for Photoacoustic Imaging
Source: Nat Commun. 2026 Oct 12;17:11562. doi: 10.1038/s41467-026-78613-7 (PMC13748559; doi:10.1038/s41467-026-78613-7)
Supplement: Supplementary file 4 [file 41467_2026_78613_MOESM5_ESM.pdf]

nature portfolio

Peer Review File

Short Wavelength Infrared Diimmonium Nano-Agents for Photoacoustic Imaging

Corresponding Author: Professor Yu Cai

This file contains all reviewer reports in order by version, followed by all author rebuttals in order by version.

Version 0:

Reviewer comments:

Reviewer #1

(Remarks to the Author)
In this work, the authors, for the first time, applied the photoacoustic contrast agent DB·2TFSI operating in the NIR-IIb region for in vivo PAI. With its optical absorption peak located at 1700 nm, DB·2TFSI overcomes the constraint of shallow penetration depth in conventional PAI, fills the research gap in NIR-IIb photoacoustic contrast agents, and provides a novel paradigm for high-resolution deep-tissue imaging, thus demonstrating substantial innovation and significant scientific value. I highly recommend this manuscript for publication in Nature Communications after some revisions.
1. In the current research on contrast agents, many researchers are engaged in fluorescence imaging studies. In the field of NIR-IIb fluorescence imaging, a series of organic/inorganic fluorescent contrast agents with outstanding performance in terms of imaging depth, signal-to-noise ratio, and other aspects have been developed. In the introduction section, the author should focus more on comparing with other imaging methods such as fluorescence imaging, to highlight the potential advantages and clinical application possibilities of PAI.
2. The authors obtained an energy gap of approximately 0.95 eV for DB·2X through DFT calculations, and subsequently concluded that the extremely small energy gap is associated with NIR-II absorption. However, the authors did not explain the role of the three anions in the parent DB molecule here. Please specifically analyze why these three anions reduce the energy gap? What is the energy gap value of the DB molecule in its non-ionic state? In addition, what insights can this anion-π conjugate system provide for the design of PA contrast agents in the future?
3. At the end of Figure 1, the authors mentioned the total interaction energy between DB and the anions. The conclusions reveal that the two anions, BOB^- and $TFSI^-$, exhibit a larger interaction energy with the DB cation, indicating a stronger anion-π^+ interaction. What impact does a larger interaction energy have on the anion-π^+ interaction? Does this impact ultimately exert any effect on light absorption?
4. Please supplement the information on the ^1H NMR spectrum, ^{13}C NMR spectrum, and HRMS data of DB·2X.
5. It is recommended to supplement the long-term stability test data of DB·2TFSI NPs to ensure the reproducibility of the experimental results and avoid potential imaging effects impairment or latent toxicity caused by in vivo aggregation.
6. The biosafety evaluation only involves short-term cytotoxicity and histological HE staining of organs. It is suggested to supplement long-term toxicity experiments: after consecutive tail vein injection for 7 days/14 days/21 days, detect the blood routine, liver function indicators, and renal function indicators of mice.
7. Since there is no specific targeting molecule, the DB·2TFSI enrichment is relatively low in the tumor PAI. It is recommended that the authors perform specific targeting modifications to increase the drug enrichment concentration.

Reviewer #2

(Remarks to the Author)
The authors expand on their prior work with N,N,N',N'-tetrakis[4-(dibutylamino)phenyl]-p-phenylenediamine. This class of molecules is not particularly novel. Electrochromic materials have been described. Nanoparticle precipitates of dyes have also obviously been described.
They previously published on this molecule: https://advanced.onlinelibrary.wiley.com/doi/full/10.1002/adhm.202301116.
There are also other NIR-2 probes, e.g., https://pubs.rsc.org/en/content/articlehtml/2021/cb/d0cb00225a
I do think there is enough novelty here to warrant publication in this journal… primarily because the large shift to 1700 nm.
1. Water absorbs strongly at 1700 nm. How does this compromise image quality?
2. IT seems like this molecule is N,N,N',N'-tetrakis[4-(dibutylamino)phenyl]-p-phenylenediamine. If so make that explicitly

clear.
3. Some more details on laser pulse width and intensity are needed.
4. Figure 4/5 images need an intensity bar. What d the colors represent? Depth, intensity, etc.?
5. The inset images need a scale bar.
6. Figure 5 needs pre-injection images to understand the contribution of background signal.
7. How was injection dose optimized?
8. What is the zeta potential of these aggregates?
9. The methods section do not describe how to the particles are formed.
10. Some discussion of these aggregates (stability and formation) in light of solvent are needed.
a. Do they break apart in DMSO etc.?
b. What salt concentrations are needed?
c. Can they form in the presence of albumin?
d. What is on their surface when you inject them.
11. What is the circulation time of these materials?

Version 1:

Reviewer comments:

Reviewer #1

(Remarks to the Author)
The manuscript is well revised and can be accepted now.

Reviewer #3

(Remarks to the Author)
In this manuscript, the authors report a series of diimmonium-based nano-agents, DB·2X, for photoacoustic imaging under 1700 nm excitation. The extension of photoacoustic contrast agents toward the NIR-IIb region is potentially interesting, and the comparison between 1064 nm and 1700 nm excitation may provide useful information for the development of long-wavelength photoacoustic imaging probes. However, the current biological and in vivo evidence is still insufficient to fully support the strong claims regarding tumor imaging and clinical translation. In particular, the in vivo experiments mainly demonstrate lower background signal under 1700 nm excitation, but they do not yet establish disease-specific or tumor-targeted imaging capability. I therefore recommend major revision before this manuscript can be considered further.

1. The animal imaging experiments are too limited to support the broad biomedical claims. Current mice models may show that 1700 nm excitation reduces background interference, but they do not fully demonstrate general applicability for deep-tissue disease imaging. Additional in vivo models, more animals, and quantitative statistical analysis are needed. At minimum, the authors should provide clear animal numbers, biological replicates, statistical tests, and quantitative tumor-to-background or lesion-to-normal tissue ratios.
2. The claim of “deep-tissue imaging” requires more direct experimental support. Lower background signal in whole-body mouse images does not necessarily prove superior tissue penetration. The authors should include phantom experiments using tissue-mimicking materials of different thicknesses to directly compare the penetration depth and signal attenuation under 1064 nm and 1700 nm excitation. Without such data, statements about deep-tissue imaging should be toned down.
3. The effect of water absorption at 1700 nm needs to be addressed more carefully. The response to reviewer #2 states that water does not interfere with 1700 nm photoacoustic detection, but the manuscript also indicates that D_2O was used in some photothermal experiments to avoid water-related background interference. The authors should provide a more discussion of water absorption in the NIR-IIb window and explain how it affects imaging in vivo.
4. Biodistribution and clearance data are insufficient. The authors state that DB·2TFSI NPs show circulation and clearance based on longitudinal PA images, but this does not provide a complete biodistribution profile. This is particularly important as nanoparticles without active targeting may accumulate in the liver, spleen, kidney, or other reticuloendothelial organs.
5. The biosafety evaluation remains preliminary. The authors should clarify the number of injections, total administered dose, animal number, and whether body weight, behavior, and organ coefficients were monitored. If comprehensive long-term toxicity data are not available, the claims about clinical translatability should be weakened.
6. The manuscript should distinguish more clearly between wavelength advantage and biological targeting advantage. The current data support the idea that 1700 nm excitation can reduce endogenous background signal compared with 1064 nm excitation. However, the data do not prove that DB·2TFSI NPs can specifically identify lesions or tumors. This distinction should be clearly stated throughout the manuscript.
7. There appears to be inconsistency in the description of the tumor model. In the results section, the authors refer to a subcutaneous xenograft tumor model, whereas the methods section mentions an orthotopic pancreatic cancer mouse model. The authors should carefully check and correct the model description.
8. Some claims in the abstract and conclusion are overstated. Phrases such as “clinical translatability,” “precision-guided therapies,” “early disease detection,” and “revolutionize non-invasive diagnostics” are not sufficiently supported by the current data.

Version 2:

Reviewer comments:

Reviewer #3

(Remarks to the Author)
The authors have properly addressed my questions and I don't have any other comments.

Open Access This Peer Review File is licensed under a Creative Commons Attribution 4.0 International License, which permits use, sharing, adaptation, distribution and reproduction in any medium or format, as long as you give appropriate credit to the original author(s) and the source, provide a link to the Creative Commons license, and indicate if changes were made.
In cases where reviewers are anonymous, credit should be given to 'Anonymous Referee' and the source.
The images or other third party material in this Peer Review File are included in the article's Creative Commons license, unless indicated otherwise in a credit line to the material. If material is not included in the article's Creative Commons license and your intended use is not permitted by statutory regulation or exceeds the permitted use, you will need to obtain permission directly from the copyright holder.
To view a copy of this license, visit https://creativecommons.org/licenses/by/4.0/

Reviewer #1 (Remarks to the Author):

In this work, the authors, for the first time, applied the photoacoustic contrast agent DB·2TFSI operating in the NIR-IIb region for in vivo PAI. With its optical absorption peak located at 1700 nm, DB·2TFSI overcomes the constraint of shallow penetration depth in conventional PAI, fills the research gap in NIR-IIb photoacoustic contrast agents, and provides a novel paradigm for high-resolution deep-tissue imaging, thus demonstrating substantial innovation and significant scientific value. I highly recommend this manuscript for publication in Nature Communications after some revisions.

1. In the current research on contrast agents, many researchers are engaged in fluorescence imaging studies. In the field of NIR-IIb fluorescence imaging, a series of organic/inorganic fluorescent contrast agents with outstanding performance in terms of imaging depth, signal-to-noise ratio, and other aspects have been developed. In the introduction section, the author should focus more on comparing with other imaging methods such as fluorescence imaging, to highlight the potential advantages and clinical application possibilities of PAI.

Response: We thank the reviewer for the suggestion. Optical imaging is of great importance in biomedicine. The development of fluorescence imaging has provided excellent spatiotemporal resolution for non-invasive in vivo imaging. Consequently, numerous researchers have devoted efforts to the study of fluorescence imaging in the near-infrared (NIR) region. However, the tissue penetration depth and spatial resolution of in vivo fluorescence imaging are severely limited by various factors, including the sensitivity of imaging equipment, autofluorescence interference from tissues, light scattering and absorption effects in biological tissues, and the quantum yield of fluorescent probes. Furthermore, even if fluorescent probes with NIR absorption can be prepared, it is often difficult to achieve a large Stokes shift, and their fluorescence emission can hardly be tuned to the NIR-IIb window, ultimately failing to realize high-resolution fluorescence imaging in deep tissues. Photoacoustic imaging combines the advantages of optical imaging and ultrasound. Ultrasonic signals are much less scattered in tissues than photons, thus enabling effective deep-tissue imaging while maintaining high resolution. Meanwhile, three-dimensional photoacoustic imaging facilitates the precise localization of lesions. Since it does not rely on fluorescence emission, photoacoustic agents are free from photobleaching and are barely affected by tissue autofluorescence. In photoacoustic imaging, pulsed lasers serve as the excitation light source with low energy, causing less damage to tissues and better meeting the developmental requirements of clinical imaging.

2. The authors obtained an energy gap of approximately 0.95 eV for DB·2X through DFT calculations, and subsequently concluded that the extremely small energy gap is associated with NIR-II absorption. However, the authors did not explain the role of the three anions in the parent DB molecule here. Please specifically analyze why these three anions reduce the energy gap? What is the energy gap value of the DB molecule in its non-ionic state? In addition, what insights can this anion-π conjugate system provide for the design of PA contrast agents in the future?

Response: The non-ionic DB molecule is an aromatic ring system containing multiple tertiary amines. The amine nitrogen atoms possess electron-donating ability, which results in a high

electron density on the aromatic rings, forming an electron-rich aromatic ring system. Upon oxidation, the nitrogen atoms in the central aromatic ring lose electrons to form cationic radical aromatic rings, whereupon the aromatic rings transform from an electron-rich state to an electron-deficient state. As counterions, the three types of anions bind through electrostatic interactions to form stable ion pairs, ultimately maintaining charge balance and structural stability. The LUMO is characterized by electron deficiency and partial positive charge. Therefore, the introduction of the three anions stabilizes the electron-deficient LUMO, leading to a significant decrease in the energy of the LUMO. In contrast, the HOMO is an occupied orbital with fully filled electrons, and the anions exert a repulsive effect on the electrons in the HOMO; thus, the energy of the HOMO remains essentially unchanged. Consequently, the energy gap is ultimately reduced. As shown in Table 1 of the manuscript, the energy gap of the non-ionic DB molecule was calculated to be 3.79 eV through theoretical computations. Firstly, for PA contrast agents with an anion-π conjugate system, the interaction between the anion and π-system can significantly narrow the HOMO–LUMO energy gap, red shifting the absorption peak to the NIR-II window or even near the NIR-IIb window, thereby meeting the requirements of penetration depth and signal-to-noise ratio for deep-tissue imaging. Secondly, the introduction of anions can effectively expand the intermolecular movement space and enhance vibrational relaxation, resulting in a greater proportion of the excited-state energy returning to the ground state through non-radiative transitions. This facilitates the conversion of photon energy into thermal effects, which further generate photoacoustic signals via thermal expansion. Furthermore, unlike the complex donor-acceptor (D-A) structures that require elaborate synthesis, the anion-π conjugate system can achieve a low energy gap, strong NIR absorption, and enhanced non-radiative transition capability simply by regulating the oxidation state. Therefore, the anion-π conjugate system is bound to occupy a prominent position in the design paradigm of future PA contrast agents.

3. At the end of Figure 1, the authors mentioned the total interaction energy between DB and the anions. The conclusions reveal that the two anions, BOB^- and $TFSI^-$, exhibit a larger interaction energy with the DB cation, indicating a stronger anion-π^+ interaction. What impact does a larger interaction energy have on the anion-π^+ interaction? Does this impact ultimately exert any effect on light absorption?

Response: The larger the total interaction energy of the anion−π system, the stronger the interaction between the anion and the electron-deficient aromatic ring, and the more stable their binding will be. A larger total interaction energy can significantly stabilize the LUMO orbital of the molecule, further narrowing the HOMO-LUMO energy gap, thereby enabling the absorption wavelength to redshift to the NIR-II region

4. Please supplement the information on the ^{1}H NMR spectrum, ^{13}C NMR spectrum, and HRMS data of DB·2X.

Response: We have successfully obtained single crystal X-ray diffraction data for three types of DB·2X (X=$TFSI^-$, BF_4^-, BOB^-), and this technique has also confirmed the molecular structure:

clarifying the connection mode between the parent and cation, the stoichiometric ratio of cation to each anion, and the identities of $TFSI^-$, BF_4^-, and BOB^- anions in the lattice; The supplementary HRMS (positive ion ESI mode) confirmed that the theoretical m/z of the cation core: single charged ion $C_{62}H_{92}N_6^+$ is 920.74, and the measured values are DB·2TFSI (920.7432), $DB{\cdot}2BF_4$ (920.7433), and DB·2BOB (920.7433), respectively. At the same time, the theoretical m/z of the double charged ion $C_{62}H_{92}N_6^{2+}$ corresponding to the main peak is 460.37, and the measured values are DB·2TFSI (460.3710), $DB{\cdot}2BF_4$ (461.3773), and DB·2BOB (460.8746) confirmed the integrity of the organic framework. Due to the wide nuclear magnetic resonance peak shape of diimide salt compounds, there are no attributable signal peaks. Therefore, we conducted alternative experiments using Fourier transform infrared spectroscopy (FT-IR). FT-IR further confirmed the presence of various anions through characteristic vibrational fingerprints: the S=O/C-F stretching peak of DB·2TFSI, the B-F stretching peak of $DB{\cdot}2BF_4$, and the C=O/C-O/B-O stretching peak of DB·2BOB are consistent with the C-H stretching peak of the butyl chain at 2950-2850 cm^{-1} in all salts. The combination of final single crystal X-ray diffraction (definitive structural proof), HRMS (quality verification), and FT-IR (functional group verification) fully meets the requirements for structural characterization.

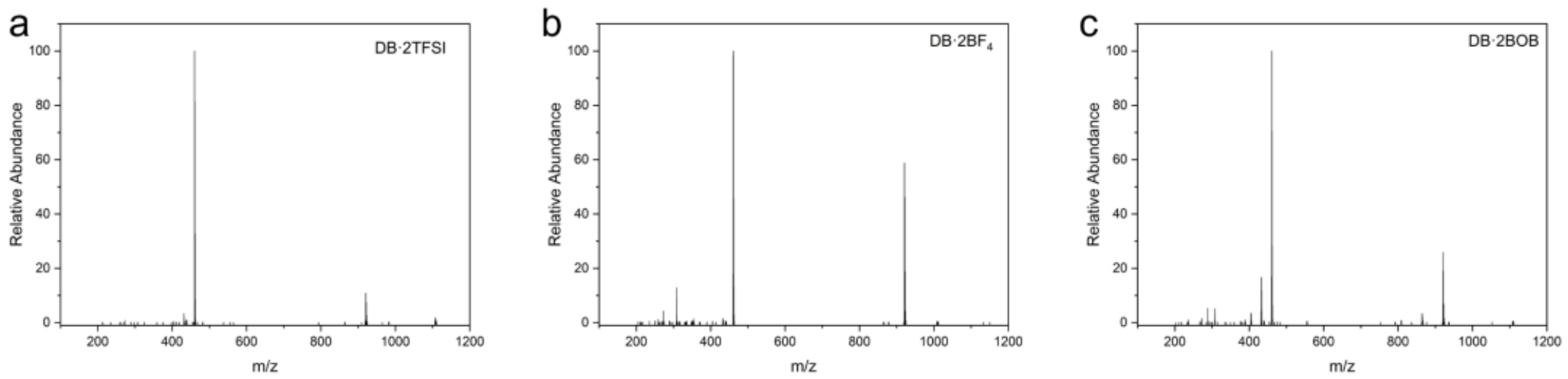

(a-c) Total ion chromatogram in the positive ESI mode of the DB·2X (X=$TFSI^-$, BF_4^-, BOB^-).

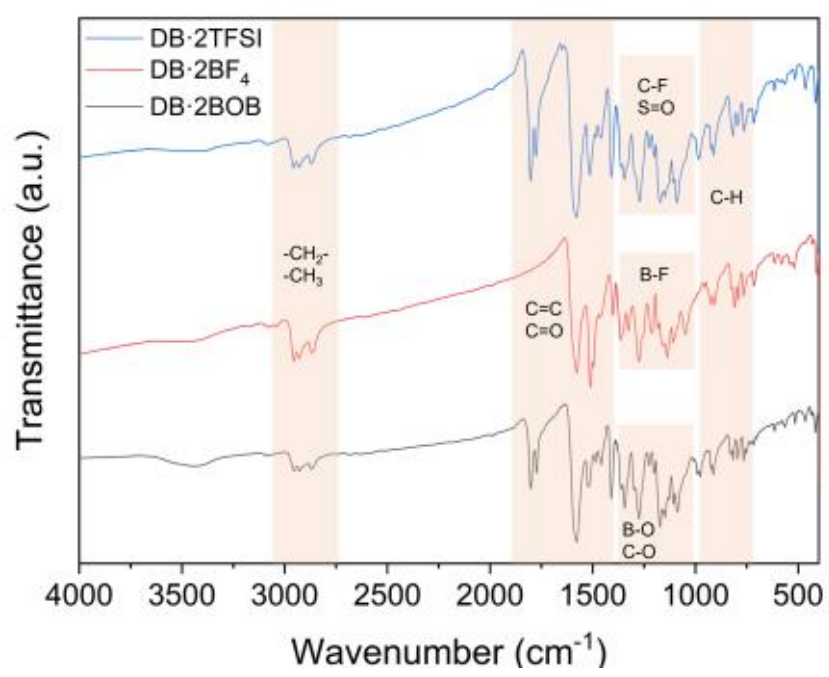

FT-IR spectra of DB·2TFSI (blue), $DB{\cdot}2BF_4$ (red) and DB·2BOB (black).

5. It is recommended to supplement the long-term stability test data of DB·2TFSI NPs to ensure the reproducibility of the experimental results and avoid potential imaging effects impairment or latent toxicity caused by in vivo aggregation.

Response: Thank you to the reviewer for comment. We have supplemented the long-term stability data of DB·2X NPs over 12 days. All three NPs (DB·2TFSI NPs, $DB{\cdot}2BF_4$ NPs,

DB·2BOB NPs) maintain a stable hydrodynamic diameter of ~100 nm from Day 1 to Day 12, with no aggregation observed. This confirms excellent colloidal stability, ensuring reproducible results and avoiding in vivo aggregation-related risks.

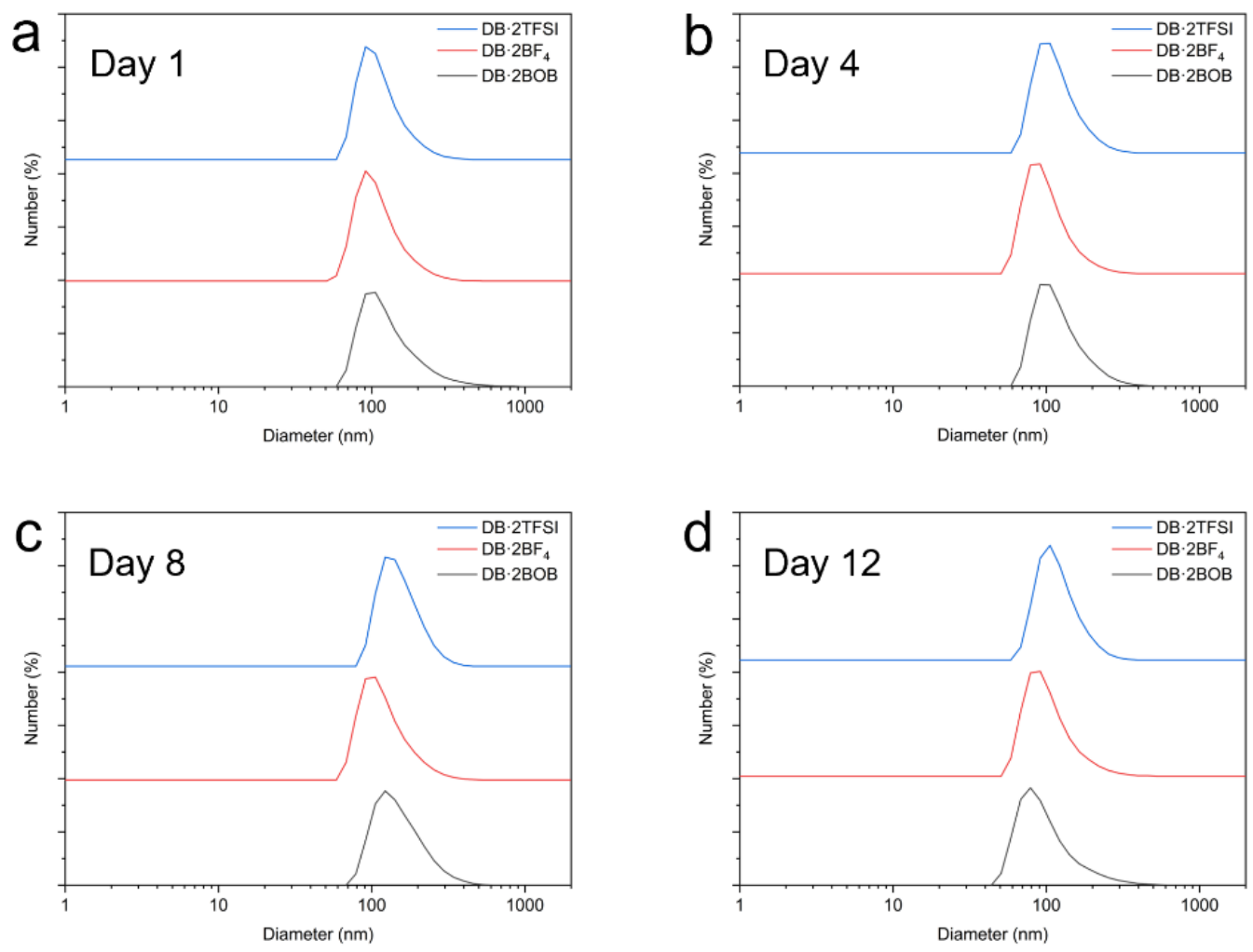

(a-d) Particle size of DB·2X (X=TFSI$^-$, BF_4^-, BOB$^-$) on different days.

6. The biosafety evaluation only involves short-term cytotoxicity and histological HE staining of organs. It is suggested to supplement long-term toxicity experiments: after consecutive tail vein injection for 7 days/14 days/21 days, detect the blood routine, liver function indicators, and renal function indicators of mice.

Response: We appreciate the reviewer's suggestion on biosafety evaluation. To further verify the long-term biocompatibility of our NPs, we performed long-term toxicity studies in mice after tail vein injections. Blood routine, liver function, and renal function indexes were analyzed at 7, 14, and 21 days. All detected the blood routine, liver function indicators, and renal function indicators of mice remained within normal ranges.

Blood routine test of mice at different days after injection of DB·2TFSI NPs.

Test items	DAY 7 (n=3)			DAY 14 (n=3)			DAY 21 (n=3)		
WBC	5.13	4.87	4.88	4.92	4.85	8	4.95	4.68	4.39
RBC	7.94	9.18	9.7	10.58	9.93	10.15	10.3	10.74	8.79
HGB	147	152	164	194	162	175	199	203	152
MCV	47.8	51	52.1	53.4	49.5	51.5	54.7	55.1	52.4
PLT	498	526	555	533	560	526	433	601	457
Lym#	3.39	3.29	3.21	3.43	3.5	4.62	3.38	1.97	2.96
Mid#	0.33	0.28	0.31	0.35	0.33	0.51	0.31	0.39	0.25
GR#	1.41	1.3	1.36	1.14	1.02	2.87	1.26	2.32	1.18
Lym%	66.1	67.5	65.8	69.7	72.1	57.8	68.2	42.2	67.4
Mid%	6.4	5.8	6.3	7.2	6.8	6.4	6.3	8.3	5.7
GR%	27.5	26.7	27.9	23.1	21.1	35.8	25.5	49.5	26.9

P_LCR	56.4	56.8	34.6	54	28.1	32.7	62.7	13.8	37
P_LCC	281	299	192	288	157	172	272	83	169
HCT	38	46.8	50.5	56.5	49.2	52.2	56.3	59.2	46.1
MCHC	387	325	325	343	329	335	353	343	330
RDW_CV	12.8	10.9	11.8	12.8	11.2	11.7	13.4	13.6	11.6
RDW_SD	66.6	60.8	61	60.8	57.1	63.3	62.7	66.1	57.4
MCH	18.5	16.6	16.9	18.3	16.3	17.2	19.3	18.9	17.3
MPV	9	9.2	9.5	9.6	9.1	9.3	9.7	9.7	9.4
PCT	0.448	0.484	0.527	0.512	0.51	0.489	0.42	0.583	0.43
PDW	17.1	17.1	17.6	16.8	17.1	16.8	16.8	17.7	18

Liver function indicators and renal function indicators of mice at different days after injection of DB·2TFSI NPs.

**Test items**	**DAY 7 (n=3)**			**DAY 14 (n=3)**			**DAY 21 (n=3)**		
ALT (U/L)	34.7	31.9	28.0	39.6	38.7	31.8	35.5	37.5	38.8
AST (U/L)	105.4	101.2	108.5	186.5	174.1	100.0	210.3	206.3	165.6
CREA (umol/L)	12.2	14.6	16.0	18.4	18.1	13.4	15.9	15.8	16.0
BUN (mmol/L)	2.519	2.747	2.752	2.346	2.318	2.519	3.866	3.265	2.500

7. Since there is no specific targeting molecule, the DB·2TFSI enrichment is relatively low in the tumor PAI. It is recommended that the authors perform specific targeting modifications to increase the drug enrichment concentration.

Response: Thank you to the reviewer for comment on improving the enrichment efficiency of DB·2TFSI NPs in tumor PAI through specific targeted modifications. The core focus of this study is the molecular design of anion - π conjugated systems and the mechanism of PAI action, with a focus on breaking through the bottleneck of PAI technology in the NIR-IIb window, aiming to achieve high spatial resolution and high signal-to-noise ratio in ultra deep tissue imaging. At present, we have innovatively validated the deep tissue PAI ability of the reagent under the NIR-IIb window in this study. We fully agree that targeted modification is an effective strategy to enhance the enrichment concentration of DB·2TFSI NPs in tumor tissues. However, specific tumor targeting research involves multiple complex and interrelated steps, including screening and synthesis of personalized target molecules (such as antibodies, peptides, or small molecule ligands), successful coupling of target molecules with photoacoustic contrast agents, evaluation of potential toxicity and off target effects in vivo, as well as the potential impact of target introduction on the aggregation behavior and photophysical properties of DB·2TFSI NPs. These may further interfere with its PAI performance. Therefore, specific targeted modification experiments belong to a relatively independent and systematic research work. Due to the current research cycle and experimental conditions, we have not yet conducted targeted design and related experiments. It should be noted that the results obtained in this study can fully support the unique advantages exhibited by anionic - π conjugated system molecules in the field of deep PAI. In the subsequent in-depth PAI related research, we will focus on the importance of active targeting in this field, screen target molecules with universality for surface modification of NPs,

establish relatively complete design principles and carry out corresponding experimental verification, and further improve the application value of this system.

Reviewer #2 (Remarks to the Author):

The authors expand on their prior work with N,N,N',N'-tetrakis[4-(dibutylamino)phenyl]-p-phenylenediamine. This class of molecules is not particularly novel. Electrochromic materials have been described. Nanoparticle precipitates of dyes have also obviously been described. They previously published on this molecule: https://advanced.onlinelibrary.wiley.com/doi/full/10.1002/adhm.202301116. There are also other NIR-2 probes, e.g., https://pubs.rsc.org/en/content/articlehtml/2021/cb/d0cb00225a. I do think there is enough novelty here to warrant publication in this journal… primarily because the large shift to 1700 nm.

1. Water absorbs strongly at 1700 nm. How does this compromise image quality?

Response: We thank the reviewer for this comment. Wang et al. (*Nat. Nanotechnol.* 2022, 17, 653–660) and Kong et al. (*Nat. Commun.* 2023, 14, 1079) demonstrated that the absorbance of water at 1700 nm is relatively low. Although water has a certain degree of absorption, in PAI, water can be excluded from the signal as a background, so there is no interference from water in the actual 1700 nm photoacoustic detection. In our PAI measurements, we further confirmed that the background signal from water at 1700 nm is negligible compared with the PA signal of our DB·2TFSI NPs. These results demonstrate that the absorbance of water at 1064 nm or 1700 nm does not compromise the imaging quality of our system. Due to the extremely low optical absorption coefficient of water at both wavelengths, it fails to capture sufficient photon energy. Consequently, the limited absorbed photon energy cannot be converted into effective thermal energy, which ultimately results in insufficient thermal expansion effect to generate acoustic waves—thus no photoacoustic signal is produced.

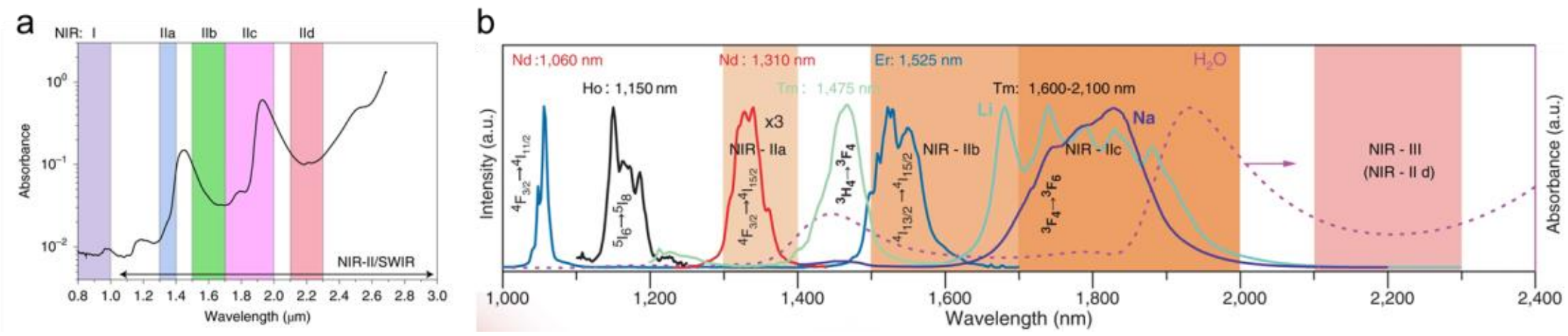

(a). Absorption spectra of water in the NIR-II and NIR-III regions[1]. Copyright 2022, Nature Publishing Group. (b). Absorption spectra of water in the NIR-II and NIR-III regions (Purple dashed line)[2]. Copyright 2023, Nature Publishing Group.

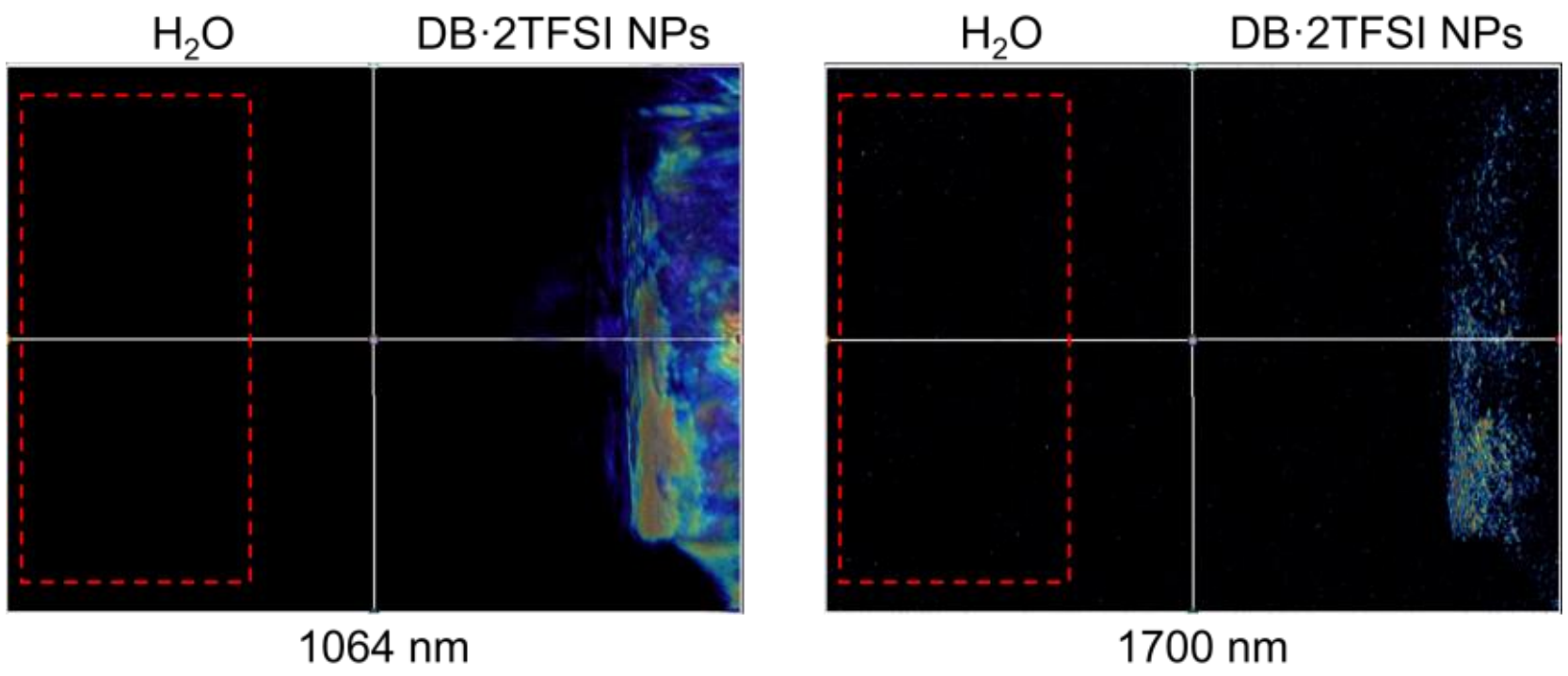

PA signal profile of water and 1 mM DB·2TFSI NPs excited by a 1700 nm pulsed laser.

2. IT seems like this molecule is N,N,N',N'-tetrakis[4-(dibutylamino)phenyl]-p-phenylenediamine. If so make that explicitly clear.

Response: We sincerely appreciate the reviewer's comment pointing out the molecular nomenclature issue. In response to this comment, we have explicitly corrected the molecular name in the methods section of the main text: replacing N,N,N',N'-tetra[4-(dibutylamino)phenyl]-1,4-phenylenediamine with the accurate N,N,N',N'-tetrakis[4-(dibutylamino)phenyl]-p-phenylenediamine to ensure clarity and accuracy.

3.Some more details on laser pulse width and intensity are needed.

Response: We thank the reviewer for this comment. The detailed parameters of the parametric generation laser used in our PAI experiments are:

Max output: 350 mJ per pulse;

Pulse duration: 3–6 ns;

Wavelength: 660–2600 nm.

4.Figure 4/5 images need an intensity bar. What d the colors represent? Depth, intensity, etc.?

Response: We thank the reviewer for the suggestion. We have added an intensity bar (color bar) to Figures 4 and 5. The color scale represents the PA signal intensity of our DB·2TFSI NPs: brighter colors indicate higher PA signal intensity, while darker colors indicate lower intensity (not depth). The updated figures are included in the revised manuscript.

5.The inset images need a scale bar.

Response: We thank the reviewer for this suggestion. We have added a scale bar in the illustration and updated the length of the scale bar in the figure. The revised images are now

included in the manuscript.

6. Figure 5 needs pre-injection images to understand the contribution of background signal.

Response: We thank the reviewer for this comment. The 0 h time-point images in Figure 5 were acquired before intravenous injection of DB·2TFSI NPs, and thus serve as the pre-injection control images to evaluate the background signal. At 1064 nm, the 0-h image shows a clear PA signal originating from endogenous hemoglobin and other signals in the mouse vascular system, rather than signals from DB·2TFSI NPs. At 1700 nm, the 0-hour image shows negligible background PA signal, confirming that the interference of intrinsic tissue absorption is minimal at this wavelength.

7. How was injection dose optimized?

Response: We have comprehensively considered the performance of DB·2TFSI NPs in vitro PA imaging, animal intravenous safety guidelines, and in vivo applicability, and optimized the injection dose. In vitro PAI experiments showed strong photoacoustic signals at 1 mM. According to the recognized safe volume standard for mouse tail vein, the safe intravenous injection dose for mice is set at 5 mL/kg, with a safety upper limit of 10 mL/kg. For mice weighing 22-25 g, the injection dose is 200 μL, equivalent to a dose of 12 mg/kg. This dose ensures sufficient PA signal and good in vivo imaging biosafety.

8. What is the zeta potential of these aggregates?

Response: Thank you for the reviewer's comment. We have supplemented the Zeta potential data (n=3) of three types of DB·2X NPs aggregates (DB·2TFSI NPs, DB·2BF_4 NPs, DB·2BOB NPs).

DB·2TFSI NPs: The average Zeta potential is -7.09±0.8 mV (individual values: -6.33 mV, -7.12 mV, -7.83 mV).

DB·2BF_4 NPs: The average Zeta potential is -6.69±0.6 mV (individual values: -6.23 mV, -6.64 mV, -7.21 mV).

DB·2BOB NPs: The average Zeta potential is -7.85±0.5 mV (individual values: -7.45 mV, -7.76 mV, -8.34 mV).

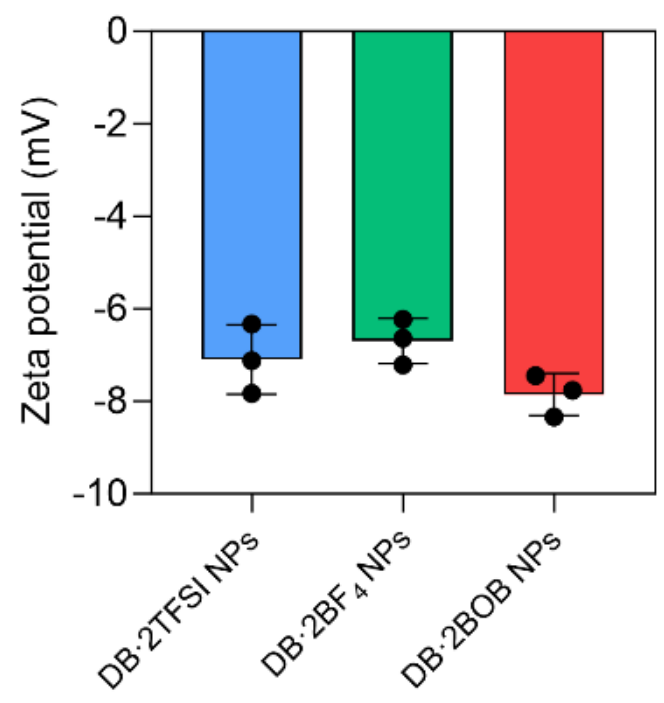

Zeta potential size of DB·2X (X=TFSI$^-$, BF_4^-, BOB$^-$).

All three types of nanoparticle aggregates exhibit negative Zeta potential, which is beneficial for maintaining colloidal stability in physiological environments and avoiding non-specific aggregation in vivo. These data have been added to the "Morphological characterization of DB·2X nano-agents" section of the manuscript.

9. The methods section do not describe how to the particles are formed.

Response: We appreciate the reviewer's comment regarding the description of nanoparticle formation. The formation method of DB·2TFSI NPs has been explicitly described in the "Synthesis of DB·2X NPs" subsection of the experimental methods section. The detailed formation process is as follows: DB·2X (1 mg) was first dissolved in THF (2 mL), and the resulting solution was slowly dripped into an aqueous solution of DSPE-PEG$_{2000}$-COOH (5 mL, 1 mg mL^{-1}). The mixture was then exposed to air and stirred overnight at room temperature on a magnetic stirrer with a stirring speed of 700 rpm, allowing tetrahydrofuran to evaporate naturally. Finally, the DB·2TFSI NPs solution with a concentration of 200 μg mL^{-1} was obtained.

10. Some discussion of these aggregates (stability and formation) in light of solvent are needed.

a. Do they break apart in DMSO etc.?

Response: Yes, the DB·2X NPs break apart in DMSO. In aqueous solution, the DB·2X NPs have a uniform hydrodynamic diameter of ~100 nm (Figure 3d and Figure S4). In DMSO, the particle size distribution shifts to much larger values. This indicates that the structure of DB·2X NPs is prone to decomposition in this polar non proton solvent.

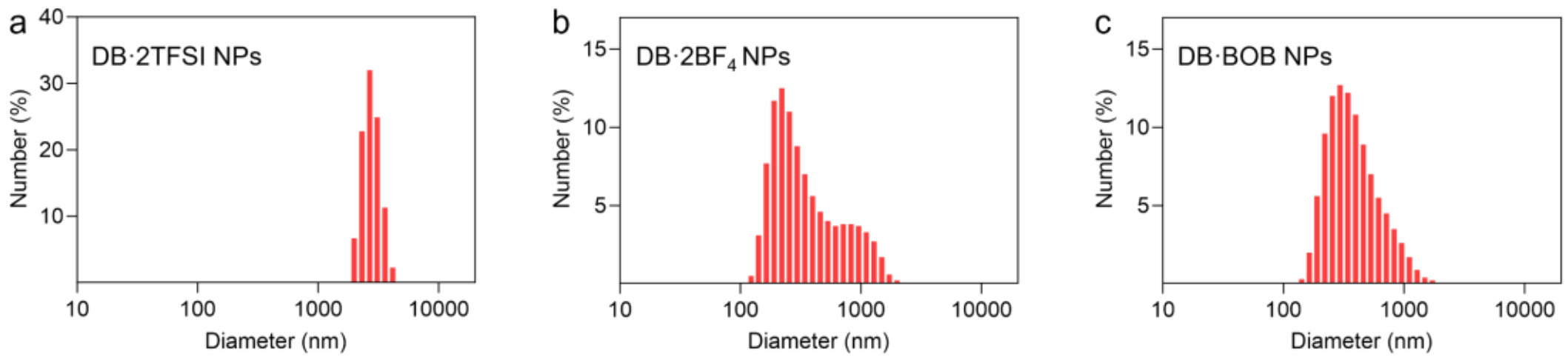

(a-c) Size Distribution of DB·2X NPs (X=TFSI, BF_4, BOB) in DMSO.

b. What salt concentrations are needed?

Response: A molar ratio of 1:2 (DB: salt anion) is required.

c. Can they form in the presence of albumin?

Response: Yes, DB·2X molecules can form in the presence of albumin. We prepared DB·2X NPs coated with albumin by solvent evaporation method and characterized their particle size and Zeta potential. The results confirmed that DB·2X molecules can effectively aggregate and form stable NPs in the presence of albumin. The relevant experiments are shown below.

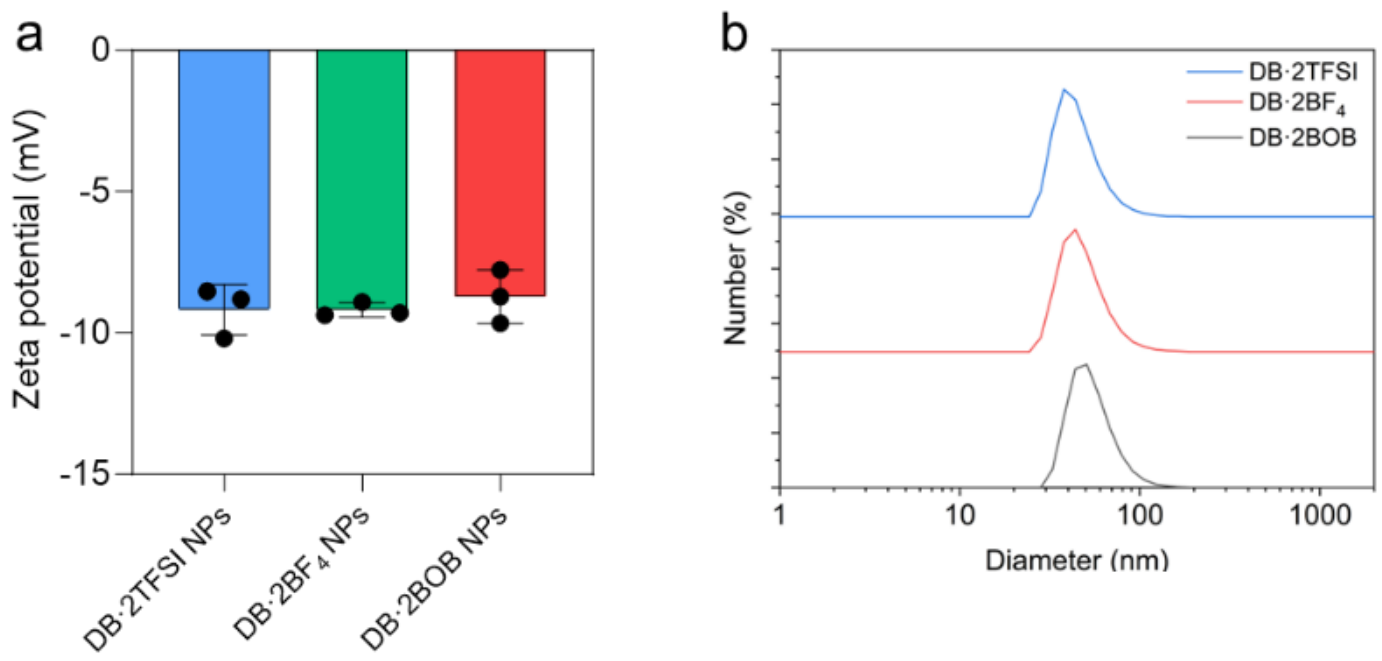

(a) Zeta potential (n=3) of DB·2X NPs (X=TFSI, BF_4, BOB) (b) Particle size distribution of DB·2X NPs.

d. What is on their surface when you inject them.

Response: When DB·2TFSI NPs are injected in vivo, the surface of the NPs is modified with DSPE-PEG_{2000}-COOH.

11.What is the circulation time of these materials?

Response: The circulation time of DB·2TFSI NPs has been verified in Figure 5a of this study. After tail vein injection, the photoacoustic images excited by a pulsed laser at 1700 nm show that DB·2TFSI NPs are distributed throughout the body at 4–6 h post-injection. Subsequently, the photoacoustic intensity of DB·2TFSI NPs gradually decreases after 8 h, and almost no photoacoustic signal of DB·2TFSI NPs is observed at 24 h post-injection. Based on the above results, the circulation time of DB·2TFSI NPs in vivo is approximately 24 h, with the peak distribution at 4–6 h and gradual clearance thereafter.

References

1 Wang, F. *et al.* In vivo non-invasive confocal fluorescence imaging beyond 1,700 nm using superconducting nanowire single-photon detectors. *Nature Nanotechnology* **17**, 653-660 (2022). https://doi.org:10.1038/s41565-022-01130-3

2 Chang, Y. *et al.* Bright Tm3+-based downshifting luminescence nanoprobe operating around 1800 nm for NIR-IIb and c bioimaging. *Nature Communications* **14**, 1079 (2023). https://doi.org:10.1038/s41467-023-36813-5

Reviewer #1 (Remarks to the Author):

The manuscript is well revised and can be accepted now.

Response: We are grateful to the reviewer for the recognition of our work.

Reviewer #3 (Remarks to the Author):

In this manuscript, the authors report a series of diimmonium-based nano-agents, DB·2X, for photoacoustic imaging under 1700 nm excitation. The extension of photoacoustic contrast agents toward the NIR-IIb region is potentially interesting, and the comparison between 1064 nm and 1700 nm excitation may provide useful information for the development of long-wavelength photoacoustic imaging probes. However, the current biological and in vivo evidence is still insufficient to fully support the strong claims regarding tumor imaging and clinical translation. In particular, the in vivo experiments mainly demonstrate lower background signal under 1700 nm excitation, but they do not yet establish disease-specific or tumor-targeted imaging capability. I therefore recommend major revision before this manuscript can be considered further.

1. The animal imaging experiments are too limited to support the broad biomedical claims. Current mice models may show that 1700 nm excitation reduces background interference, but they do not fully demonstrate general applicability for deep-tissue disease imaging. Additional in vivo models, more animals, and quantitative statistical analysis are needed. At minimum, the authors should provide clear animal numbers, biological replicates, statistical tests, and quantitative tumor-to-background or lesion-to-normal tissue ratios.

Response: We appreciate the reviewer's important comment. We agree that in vivo results should be interpreted as proof-of-concept evidence rather than comprehensive validation of deep tissue disease models. Therefore, we have revised the corresponding biomedical statements in the revised manuscript. We apologize that the manuscript does not clearly describe the number of animals and the concentration and dosage of

experimental drugs. Three independent animals (n=3) were used for each in vivo imaging model. Each animal received a single dose of 200 µL of 1 mM DB·2TFSI NPs. The administration routes are intravaginal administration in the uterine adhesion model and intravenous administration in the tumor model. Sequential imaging of the same animal was performed under excitation at 1064 nm and 1700 nm, allowing for paired comparison between the two wavelengths. We have modified the relevant information in the method. In addition, PA measurements in vitro were independently repeated three times (n=3). These in vitro replicates support the repeatability of wavelength and concentration dependent PA signals, presented separately from in vivo biological replicates. Therefore, the revised manuscript now describes animal experiments as preliminary proof of concept validation, demonstrating that under test conditions, endogenous background decreases and signal to background contrast increases. The statement about the universal applicability of deep tissue disease imaging has been deleted or softened. Further research is needed on more disease models and larger animal cohorts to establish universality and translation relevance.

2. The claim of "deep-tissue imaging" requires more direct experimental support. Lower background signal in whole-body mouse images does not necessarily prove superior tissue penetration. The authors should include phantom experiments using tissue-mimicking materials of different thicknesses to directly compare the penetration depth and signal attenuation under 1064 nm and 1700 nm excitation. Without such data, statements about deep-tissue imaging should be toned down.

Response: We agree with the reviewer that reduced background in whole-body PA images does not, by itself, constitute direct evidence of improved tissue penetration. Since photoacoustic imaging experiments are performed by placing the sample completely into water for 3D scanning, it is difficult for us to prepare samples wrapped in cylindrical biological tissues that are not disturbed by any bubbles, so the experiments of penetration depth are difficult to perform. We have to no longer claim that 1700 nm excitation provides superior penetration depth compared with 1064 nm

excitation. Throughout the revised manuscript, phrases such as "enhanced penetration depth" and "deep tissue imaging" have been removed or replaced with other descriptions, including "require the suppression of endogenous background signals", "reduce endogenous background", "improve signal-to-background contrast".

3. The effect of water absorption at 1700 nm needs to be addressed more carefully. The response to reviewer #2 states that water does not interfere with 1700 nm photoacoustic detection, but the manuscript also indicates that D_2O was used in some photothermal experiments to avoid water-related background interference. The authors should provide a more discussion of water absorption in the NIR-IIb window and explain how it affects imaging in vivo.

Response: We thank the reviewer for this comment. In the photothermal experiments, we measured the temperature increase of the entire solution during prolonged irradiation at 1550 nm. Water has an appreciable vibrational overtone absorption band near 1450 nm, and 1550 nm remains on the long-wavelength side of this band. Consequently, H_2O can absorb part of the incident energy and contribute to cumulative bulk heating. D_2O was therefore used to minimize solvent-derived heating and to better isolate the photothermal contribution of the DB·2TFSI NPs. In contrast, PA imaging detects transient acoustic waves generated by rapid thermoelastic expansion following nanosecond pulsed excitation. The initial PA pressure is generally related to the local optical absorption coefficient, local optical fluence, and thermoelastic conversion efficiency, as expressed by $p_0 \propto \Gamma \mu_a F$. In our experiments, the laser pulse duration was 3–6 ns. Under the same acquisition and image-reconstruction conditions, pure water did not produce a detectable PA signal comparable to that of 1 mM DB·2TFSI NPs under 1700 nm excitation. This observation indicates that water-derived PA background did not dominate DB·2TFSI NPs signal under the tested conditions. Nevertheless, this result does not mean that water absorption at 1700 nm is absent or negligible. Although the water absorption around 1700 nm is relatively lower than that near the stronger absorption band around 1450–1550 nm, water may still attenuate the excitation fluence

delivered to deeper tissues. Therefore, water absorption can influence in vivo imaging even when the directly detected water-derived PA background is weak.

4. Biodistribution and clearance data are insufficient. The authors state that DB·2TFSI NPs show circulation and clearance based on longitudinal PA images, but this does not provide a complete biodistribution profile. This is particularly important as nanoparticles without active targeting may accumulate in the liver, spleen, kidney, or other reticuloendothelial organs.

Response: We thank the reviewer for this comment. We agree that longitudinal PA imaging of the tumor region does not provide a comprehensive assessment of systemic biodistribution or clearance. The reduction in local PA signal may reflect redistribution, degradation or a concentration below the detection threshold and therefore cannot be interpreted as direct evidence of whole-body elimination. We have removed unsupported statements regarding nanoparticle circulation, metabolism, and clearance. The results are now described only as time-dependent changes in tumor-associated PA signal. We have also clarified that DB·2TFSI NPs lack an active targeting ligand and that accumulation in the liver, spleen, kidney, or other reticuloendothelial organs cannot be excluded. The histological and blood-based measurements are now explicitly described as preliminary safety assessments rather than biodistribution analyses. Quantitative organ-level distribution and excretion studies are acknowledged as necessary future work.

5. The biosafety evaluation remains preliminary. The authors should clarify the number of injections, total administered dose, animal number, and whether body weight, behavior, and organ coefficients were monitored. If comprehensive long-term toxicity data are not available, the claims about clinical translatability should be weakened.

Response: We appreciate the reviewer’s comment and agree that the current

experiments represent a preliminary safety assessment rather than a comprehensive long-term toxicological evaluation. For the proof-of-concept in vivo PA imaging experiments, DB·2TFSI NPs were administered once at a concentration of 1 mM, with a total injection volume of 400 μL per rat and 200 μL per mouse. Three for each group were used for safety assessment. Body weight, behavior, and organ coefficients were not monitored in the present imaging experiments. The current safety results are now described only as preliminary observations based on the available cellular, hematological, biochemical, and histological assessments. The terminology has been revised from “the long-term biocompatibility”, “negligible cytotoxicity” and “a long-term toxicity assay” to “the preliminary safety biocompatibility”, “low cytotoxicity” and “a short-term toxicity assay”. In future PA imaging research projects, we will pay more attention to the repeated-dose toxicity, the changes in body weight and behavior, organ coefficients, quantitative biodistribution, and longer-term biochemical outcomes of photoacoustic imaging reagents during long-term in vivo.

6. The manuscript should distinguish more clearly between wavelength advantage and biological targeting advantage. The current data support the idea that 1700 nm excitation can reduce endogenous background signal compared with 1064 nm excitation. However, the data do not prove that DB·2TFSI NPs can specifically identify lesions or tumors. This distinction should be clearly stated throughout the manuscript.

Response: We appreciate the reviewer's important comment. We agree that the wavelength dependent imaging advantage of 1700 nm excitation should be clearly distinguished from biological targeting or lesion specific recognition. DB·2TFSI NPs were PEGylated to improve its water dispersibility and colloidal stability, but not functionalized with any active targeting ligands. Therefore, current experiments have not demonstrated receptor mediated tumor targeting or disease-specific recognition. So, we have revised the inappropriate wording in the abstract, related discussions, and conclusions to clarify this distinction. We also explicitly state that the observed increase in PA signals within the tumor area should not be interpreted as evidence of tumor

specific recognition. The improved visualization obtained under 1700 nm excitation is now mainly attributed to wavelength dependent suppression of endogenous background relative to 1064 nm excitation, rather than active biological targeting of DB·2TFSI NPs. The relevant paragraphs have been marked in red.

7. There appears to be inconsistency in the description of the tumor model. In the results section, the authors refer to a subcutaneous xenograft tumor model, whereas the methods section mentions an orthotopic pancreatic cancer mouse model. The authors should carefully check and correct the model description.

Response: We thank the reviewer for this suggestion. We apologize for this inconsistency. The tumor imaging experiment was performed using a 4T1 (mouse breast cancer cells) subcutaneous tumor model, rather than an orthotopic pancreatic cancer model. The Methods section has been corrected accordingly, and all references to an orthotopic pancreatic model have been removed.

8. Some claims in the abstract and conclusion are overstated. Phrases such as "clinical translatability," "precision-guided therapies," "early disease detection," and "revolutionize non-invasive diagnostics" are not sufficiently supported by the current data.

Response: We agree that several statements in the Abstract and Conclusion exceeded the level of evidence provided by the current study. We have removed or moderated expressions including "clinical translatability," "early disease detection," "precision-guided therapies," "revolutionize non-invasive diagnostics," and "superior penetration depth." This revised manuscript validates the feasibility of DB·2TFSI NPs as a comparison platform for 1700 nm excited PA imaging. The current conclusions are limited to the investigated molecular design, optical characteristics, PA signal generation performance, and enhanced in vivo imaging contrast under the tested

experimental conditions. In future work, we will expand the scale of animal experiments to fulfill the requirements for depth validation, quantitative biodistribution analysis, targeting capability verification, and systematic toxicological evaluation.

Dear Chief Editor. Steven Lukman,

Thank you for your letter and the reviewers' comments concerning our manuscript entitled "1700 nm Diimmonium Nano-Agents for Photoacoustic Imaging". Thank you very much for your recognition of our work. We are grateful for your efforts as well as those of the reviewer.

Reviewer #3: The authors have properly addressed my questions and I don't have any other comments.

Response: Thank you very much for your careful evaluation of our work and for your valuable suggestions. We will continue to strive to conduct rigorous research.
